# Endogenous vs Exogenous Allosteric Modulators in GPCRs: A dispute for shuttling CB_1_ among different membrane microenvironments

**DOI:** 10.1038/srep15453

**Published:** 2015-10-20

**Authors:** Mariano Stornaiuolo, Agostino Bruno, Lorenzo Botta, Giuseppe La Regina, Sandro Cosconati, Romano Silvestri, Luciana Marinelli, Ettore Novellino

**Affiliations:** 1 Department of Pharmacy, University of Naples “Federico II”, via D. Montesano 49, 80131 Naples, Italy; 2 Istituto Pasteur−Fondazione Cenci Bolognetti, Dipartimento di Chimica e Tecnologie del Farmaco, Sapienza Università di Roma, Piazzale Aldo Moro 5, I-00185 Roma, Italy; 3 DiSTABiF, Seconda Università di Napoli, Via Vivaldi 43, 81100 Caserta, Italy

## Abstract

A Cannabinoid Receptor 1 (CB_1_) binding site for the selective allosteric modulator ORG27569 is here identified through an integrate approach of consensus pocket prediction, mutagenesis studies and Mass Spectrometry. This unprecedented ORG27569 pocket presents the structural features of a Cholesterol Consensus Motif, a cholesterol interacting region already found in other GPCRs. ORG27569 and cholesterol affects oppositely CB_1_ affinity for orthosteric ligands. Moreover, the rise in cholesterol intracellular level results in CB_1_ trafficking to the axonal region of neuronal cells, while, on the contrary, ORG27568 binding induces CB_1_ enrichment at the soma. This control of receptor migration among functionally different membrane regions of the cell further contributes to downstream signalling and adds a previously unknown mechanism underpinning CB_1_ modulation by ORG27569 , that goes beyond a mere control of receptor affinity for orthosteric ligands.

The endocannabinoid system comprises the GPCR family members cannabinoid receptors CB_1_ and CB_2_, their endogenous ligands (endocannabinoids) and the enzymes responsible for the synthesis and degradation of the latters^1^. Upon binding to their endogenous partial agonist anandamide or to exogenous ligands like Δ^9^-tetrahydrocannabinol, CB_1_ affects cell proliferation, motility, adhesion and apoptosis and controls a variety of physiological processes spanning from neuronal development to organs functioning^2^,^3^. Signalling by CB_1_ involves both G protein-dependent pathways, such as inhibition of adenylate cyclase, as well as G-protein independent mechanisms^4^,^5^,^6^. Due to its widespread distribution^7^ and implication in many diseases CB_1_ is ranked among the golden targets for the treatment of nausea, obesity, pain, neurodegenerative diseases and substance abuse disorders^8^.

GPCRs orthosteric binding sites have been extensively investigated to identify new ligands. Three CB_1_ ligands (Cesamet^9^, Marinol^10^, and Sativex^11^) are being prescribed to reduce chemotherapy-induced nausea, stimulate appetite or reduce pain^8^. On the contrary, the CB_1_ inverse agonist rimonabant was initially commercialized as anorectic antiobesity drug and then suspended due to its psychiatric side-effects^12^. Its withdrawal pointed out the risk of targeting GPCRs orthosteric sites, highly conserved among GPCRs^13^.

Alternative approaches for GPCRs drug discovery are thus being considered in order to develop safer drugs and achieve a better fine-tuning of GPCR functionality^14^. While orthosteric sites have faced high evolutionary pressure in order to keep an efficient binding to their endogenous ligands, the evolution of allosteric pockets has been less stringent causing their aminoacidic sequences to be poorly conserved and, as consequence, more specific for each receptor^15^. The development of functionally selective allosteric modulators is thus considered a promising avenue to develop new target specific drugs and overcome nowadays obstacles in cannabinoid-based drug discovery such as on- and off-target side effects.

To date, few compounds have been identified as exogenous CB_1_ allosteric modulators including the synthetic “ORG” compounds (ORG27569, ORG29647, ORG27759)^16^,^17^, PSNCBAM-1^18^, RTI-371^19^ and the natural endogenous modulators lipoxin A4^20^, pregnenolone^21^ and cholesterol^22^. Recently our group embarked in a Structure-Activity-Relationship (SAR) study of ORG27569^23^ which is an exquisitely selective allosteric modulator for CB_1_^23^,^24^.

Despite positively affecting CB_1_ affinity for some agonists, ORG compounds inhibit agonist-induced G-protein coupling. Independently from the CB_1_ orthosteric site being occupied or not, ORG27569 selectively hampers G-protein signalling and promotes β-arrestin2-mediated internalization of the receptor and β-arrestin1-mediated activation of kinases^17^,^25^. However, the mechanism behind CB_1_-biased signalling by allosteric ligands remains still obscure as well as the molecular basis of its selectivity over CB_2_. Furthermore, the missing identification of its binding site hampers a structure-based evolution towards new ORG27569-inspired allosteric molecules. Recently, a site partially overlapping with the CB_1_ orthosteric site has been proposed as binding pocket for ORG27569^26^. However, the proof of such hypothesis was based on a comparison between the functional activity of the wt receptor and that of mutants at the proposed binding site, while no data were shown on the effect of such mutations on the binding properties of the receptor^26^. Moreover the existence of a competition between ORG27569 and inverse agonists for the same binding site, corollary of that hypothesis, is not in line with the data proving the inability of the allosteric molecule to physically displace orthosteric ligands^24^,^27^.

Herein, through a multidisciplinary approach we physically identify an ORG27569 binding site. Interestingly, this site presents structural features of a CCM (Cholesterol Consensus Motif), a cholesterol binding region that have already been identified in other GPCRs^28^. Advanced Molecular Dynamics (MD), here presented, suggest ORG27569 binding mode and CB_1_ structural changes upon allosteric ligand binding. In cultured cells we show that, while cholesterol allows enrichment of CB_1_ at the axon, where endocannabioid pathway effectors are mainly localized^29^, ORG27569 drives CB_1_ close to the soma. This proves that the ORG27569 allosteric modulation works at least on two levels: i) by fine tuning receptor affinity for orthosteric ligands and ii) by topologically control of CB_1_ membrane localization.

## Results

### Prediction of ORG27569 candidate Binding Sites and selection of mutants

**Figure 1:**
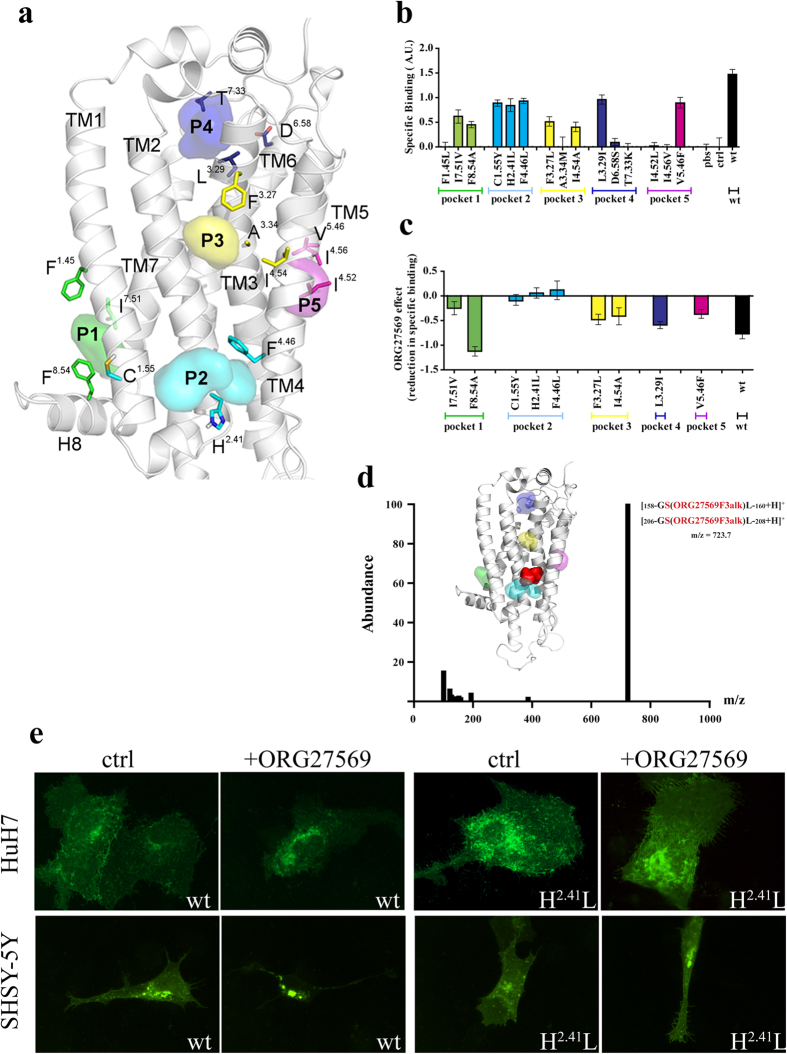
ORG27569 pocket identification. (**a**) The 5 putative allosteric pockets mapped onto the CB_1_ homology model. Probes identifying each site are represented by differently colored surfaces. The three mutated residues for each site are highlighted in colored sticks. P1 is defined by TM1-TM7 and H8 domains, P2 by TM1-4, P3 by the same TM domains of P2 but towards the extracellular region, P4 is defined by residues on TM3 and TM7, finally P5 is defined by TM3-5. (**b**) Human CB_1_wt receptor and the indicated CB_1_ mutants were transiently expressed in HEK293 cells. Membrane homogenates were obtained and T1117 binding measurement performed as described in the On line Method Sections. Specific binding correlates with the fold change increase of T1117 fluorescence in presence of AM251. (**c**) Membrane homogenates were obtained from cells expressing CB_1_wt receptor or the indicated CB_1_ mutants. Samples were incubated with ORG27569 (3 μM) for 30 minutes. T1117 specific binding measurement was performed as described above. Effect of ORG27569 treatment is expressed as change in T1117 specific binding upon ORG27569 treatment (for panels b and c the data depict the mean +/− s.e.m. and are representative of three or more independent experiments. P < 0.05. ANOVA-test was employed). (**d**) Peptides identified by LC/MS analysis and presenting **ORG27569alk3** covalently linked to S^2.45^ or to S^3.42^ of the P2 pocket. Peptide abundance is plotted as a function of mass/charge (m/z). Amino acids that could present **ORG27569alk3** covalently bound are shown in red. The inset shows the region addressed by the probe (red, surface) superimposed with the P2 binding pocket (cyan, surface); (**e**) rat CB_1_wt-GFP and CB_1_(H^2.41^L)-GFP constructs were transiently expressed in HuH7 (upper panels) and SHSY-5Y (lower panels) that were treated (+ORG27569) or not (ctrl) with ORG27569 (3 μM) for 4 hours (see also Supplementary Fig. S6).

Consensus pocket prediction on the entire CB_1_ receptor was performed to identify ORG27569 candidate binding sites. Beside the canonical orthosteric pocket, nine potential allosteric sites were identified (See Computational Protocol and Supplementary Fig. S1–3). Since ORG27569 selectively binds CB_1_ over CB_2_^23^,^24^, we only selected pockets presenting at least one aminoacidic difference between CB_1_ and CB_2_. Thus, only five potential binding sites (P1-5) for ORG27569 were further considered (Fig. 1a). With the exception of pocket 4 (P4), which partially overlaps with the orthosteric pocket, the other sites are all lipid exposed (Fig. 1a). Noteworthy, P1, P2, and P4 were previously reported as putative allosteric pocket for other GPCRs^28^,^30^,^31^. For each candidate site only 3 residues (not conserved in CB_2_) were considered for site-directed mutagenesis (Table 1). These were mutated in the corresponding CB_2_ residues rather than in Alanines, to avoid non-functional mutant receptors (see Supplementary Fig. S4 for details).

### Screening of CB1 Mutants toward ORG27569 binding pocket identification

15 CB_1_ mutants (Table 1), each carrying one CB_1_ residue substituted with the corresponding CB_2_ counterpart were generated. ORG27569 binding site was identified by testing each CB_1_ mutant in a two steps pipeline: first we *i*) excluded mutations abolishing binding to an orthosteric inverse agonist; then *ii*) we selected those mutants which affinity for orthosteric ligands was unaffected by ORG27569 treatment. As tool for the pipeline, we used a newly developed assay based on T1117, a fluorescently labeled analogue of rimonabant. We recently proved that upon binding to CB_1_, T1117 gets fluorescently quenched and that its change in fluorescence relates to the affinity of CB_1_ for orthosteric and allosteric molecules^32^. T1117 specifically bound to CB_1_wt was efficiently measured by displacement with the CB_1_ specific orthosteric ligand AM251^32^. Six of the CB_1_ mutants tested (Fig. 1b) made the CB_1_ receptor unable to bind the orthosteric ligand, thus they were tossed out. P4 partially overlaps with the T1117 binding site^32^ and T^7.33^, even if not being in direct contact with T1117, locates at the entry portal of the ligand into the orthosteric binding site^32^. Mutations on TM3 (where A^3.34^ is located) were already shown to negatively influence AM251 binding^17^ and those in the surroundings of P1 are known to abolish CB_1_ conformational changes linked to G protein activation and thus they could likely affect orthosteric binding.

The nine CB_1_ mutants still able to bind T1117 (Fig. 1b,c) moved to the second step of the pipeline. The binding of CB_1_wt to T1117 is negatively affected by ORG27569 treatment (IC_50_ = 3.0 μM)^24^,^32^. On the contrary, the three mutants C^1.55^Y, H^2.41^L and F^4.46^L, strikingly all belonging to the same P2 pocket, were completely unaffected by ORG27569, with the allosteric molecule decreasing their binding to T1117 only of a 0–10% (Fig. 1c). Mutations in pocket P3, P4 and P5 reduced the susceptibility of probe binding to ORG27569 to a lesser extent (Fig. 1c). Interesting is the effect of two mutations in the P1 pocket, where one (I^7.51^V) decreases susceptibility to ORG27569 to 70%, while another (F^8.54^A) increases the same of 30% (Fig. 1c). The effect of F^8.54^A mutation on P1 became clearer after experiments described below. Thus, *in vitro* binding measurements clearly suggest that, among the five pockets tested, P2 is a binding site for ORG27569.

### Identification of the binding site of an ORG27569-derived probe by Liquid Chromatography/Mass Spectrometry

To confirm ORG27569 addressing the P2 pocket, we converted the allosteric ligand in a molecular probe by derivatization with an alkyne moiety. Despite being extremely unreactive, alkynes can receive nucleophilic attacks from sulfhydryl or hydroxyl group of amino acids to generate covalent adducts. This extremely rare event has been shown to happen at catalytic sites of enzymes as well as in ligand binding pockets^33^.

We attempted derivatization of ORG27569 at 4 different positions (**ORG27569alk1-4**, See *Chemistry section* in the Supplementary Information). **ORG27569alk 1** and **2** were strongly insoluble and thus could not be used. **ORG27569alk4** induced massive cell detachment in within 1 hour from the treatment. On the contrary, **ORG27569alk3** was well tolerated by the cells and thus was used as candidate probe.

CB_1_-GFP expressing HEK293 cells were cultured in the presence of **ORG27569alk3**. After the treatment, CB_1_-GFP was immunopurified, digested with Proteinase K and analyzed by Liquid Chromatography/Mass Spectrometry (LC/MS). HPLC profiles of samples from **ORG27569alk3** treated cells and untreated ones were compared. These were almost totally overlapping with the exception of *i*) a fraction eluting with retention time of 14.0 min presenting the unbound probe (Supplementary Fig. S5) and *ii*) a fraction eluting with retention time of 3.8 min and present only in samples obtained from **ORG27569alk3** treated cells (Supplementary Fig. S5). The peptide eluting in this fraction presents a m/z of 723.7 Da and corresponds, with a deviation from theoretical mass (Δ mass) of 0.2 Da, to the sequence GSL (amminoacids 158–160 or 206–208) of CB_1_ (theoretical mass of the peptide 276.1 Da) presenting **ORG27569alk3** (MW = 447.8 Da) covalently linked either to S^2.45^ or to S^3.42^ (Fig. 1d) (theoretical mass [**M-H-ORG27569alk3**]^+^ of 723.9 Da). Despite the presence of nucleophilic Ser, Thr, Tyr and Cys present in the other investigated pockets (Supplementary Fig. S5), the spectra clearly indicate **ORG27569alk3** is addressing the P2 pocket, with it contacting at least S^2.45^ or S^3.42^, which both belong to P2 pocket and are in close proximity to each other (Fig. 1d).

### Effect of H^2.41^L mutation on CB_1_ intracellular localization

To finalize our ORG27569 binding pocket identification the intracellular distribution of the H^2.41^L mutant (P2) was followed in cultured cells. The mutant H^2.41^L was generated on the template of a C-terminally tagged GFP version of the rat wt receptor^34^. When expressed in Human Hepatoma Cells (HuH7), CB_1_wt-GFP appears mainly localized on the cells Plasma Membrane (PM) and in intracellular vesicles, similarly to what was already seen in many other cell type^34^ (Fig. 1d and supplementary Fig. S6). Upon treatment with ORG27569, the intracellular pool of CB_1_wt-GFP increased as already reported^17^,^24^,^25^,^27^. CB_1_(H^2.41^L)-GFP localizes at the steady state at the PM and in intracellular vesicle like the CB_1_wt protein, on the contrary the treatment with ORG27569 does not alter its localization (Fig. 1e and Supplementary Fig. S6). This data indicates that CB_1_(H^2.41^L)-GFP is correctly folded and transported to its final localization, but that it is not able to bind ORG27569, confirming our previous data indicating that P2 is a recognition pocket of ORG27569.

Finally, the CB_1_wt-GFP and the CB_1_(H^2.41^L)-GFP mutant were both expressed in cells of neuronal origin, that more closely resemble the natural context where CB_1_ is endogenously expressed. In untreated SHSY-5Y neuroblastoma cells, CB_1_wt-GFP appears mainly localized on the PM of the cells equally distributed among dendrites/axons and central body (Fig. 1e). Noteworthy, 4 hours treatment with ORG27569 moved the pool of CB_1_wt-GFP in intracellular vesicles, while CB_1_(H^2.41^L)-GFP resulted completely unaffected by the treatment (Fig. 1e). All together these data indicate that CB_1_(H^2.41^L)-GFP is not sensitive to the treatment with ORG27569 *in vitro* as well as in cultured neuronal and non neuronal cell lines. Moreover, the affinity for T1117 and the PM localization of CB_1_(H^2.41^L)-GFP indicates that the mutant is correctly folded and transported along the secretory pathway. Taken together these data confirmed P2 as an allosteric site of ORG27569 within the CB_1_ receptor.

### Theoretical predictions of CB_1_ structural changes upon ORG27569 binding

**Figure 2:**
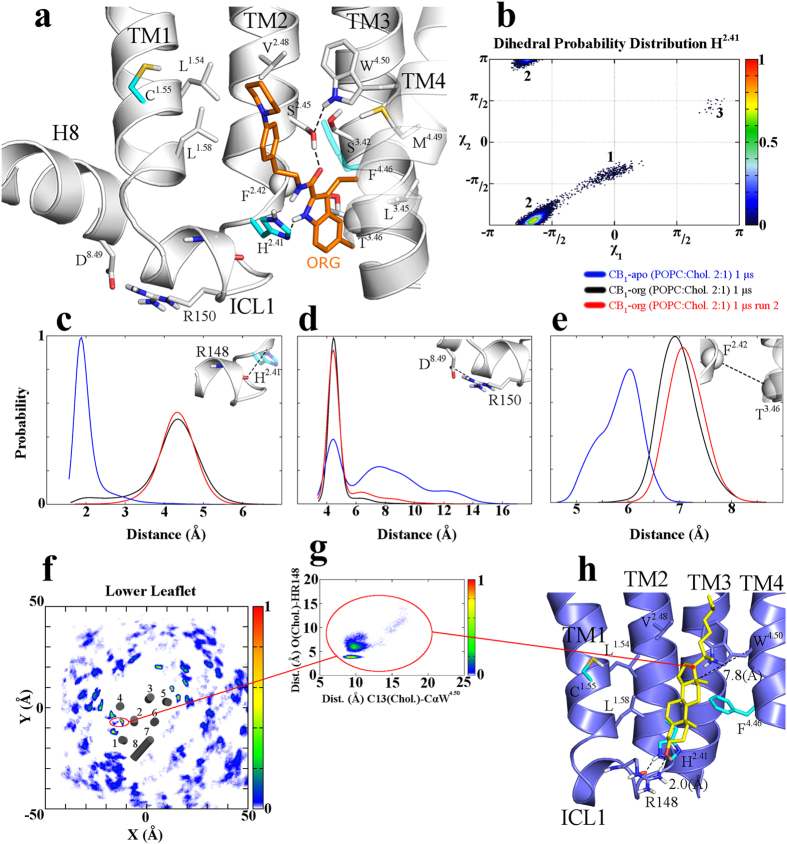
ORG27569 and cholesterol binding mode. (**a**) ORG27569 Binding mode. Key interactions included: (*i*) two H-bonds established by the 1*H*-indole-2-carboxamide group with the H^2.41^ N^δ^ atom and the S^2.45^ side chain; (*ii*) the 1-(4-ethylphenyl)piperidine arm accommodating in a hydrophobic region interacts with F^4.46^, V^2.48^, L^1.54^, and L^1.58^; and (*iii*) the 3-ethyl group plunges through a hydrophobic area establishing contacts with L^3.45^ and M^4.49^. (**b**) H^2.41^ *x*_1_ and *x*_2_ dihedral angles distribution for the CB_1_wt-ORG27569 simulation (run 1). The probability of each state was normalized. A highly represented conformer, in the CB_1_wt-ORG27569 MD, for the H^2.41^ residue (conformer 2), different from that observed in the CB_1_wt unbound state (conformer 1, see also Supplementary Fig. S9) was noticed. Remarkably, in the CB_1_wt-ORG27569 simulations, the shift from H^2.41^ conformer 1 to 2 causes the H-bond loss between the H^2.41^ Nε (TM2) and the R148 backbone oxygen (ICL1, Fig. 2d). (**c**–**e**) Probability distribution for the H^2.41^(NHε)-R148(O), F^2.42^(Cα)-T^3.46^(Cα), and R150(CZ)-D^8.49^(CG) distance atoms, for CB_1_wt (blue lines) and the CB_1_wt-ORG27569 simulations (black , and red lines), respectively. The probability of each distance was normalized. (**f**) Probability distribution of cholesterol molecules along the x and y axes with respect to the main axis of CB_1_, only cholesterol molecule into the lower leaflet of the bilayer were considered (See Appendix A in Supplementary Information for a detailed description of the calculation of the probability distribution). (**g**) Probability distribution of the cholesterol molecule, which binds to the CCM during the CB_1_wt POPC:Chol (2:1) simulations. The probability distribution distance for the W^4.50^(Cα)-CHL(C13) carbon atoms (x axis) is plotted *vs.* the probability distribution distance for the NH(Arg148)-O atoms (y axis). (**h**) Binding mode of the cholesterol molecules in P2/CCM. The cholesterol molecule interacts with L^1.54^, L^1.58^, H^2.41^, V^2.48^, L^2.52^, F^4.46^, and W^4.50^ residues, while the R148 side chain anchors the OH apical group.

Docking of ORG27569, by means of Glide software, was focused on P2 pocket and resulted in a binding mode (Fig. 2a) in line with the reported SAR^35^ (see the *Binding mode reliability* section in the Supplementary Information), the mutagenesis data (Figs 1c–e and 2a), and the CB_1_/CB_2_ selectivity profile^23^,^24^. Both H^2.41^ and F^4.46^ directly participate in ORG27569 binding and, together with V^2.48^ are not conserved in CB_2_. We did not observe a direct interaction between ORG27569 and C^1.55^, thus it might be conceivable that the introduction of the bulkier tyrosine (C^1.55^Y substitution) could hamper the ligand entry into the P2 pocket.

To support our docking-derived pose of ORG27569 and to unravel the local receptor structural changes upon its binding, extensive MD simulations for the unbound CB_1_wt, CB_1_wt-ORG27569 and CB_1_-(H^2.41^L)-ORG27569 complexes were performed as follows: (*i*) for the CB_1_wt system, three 1 μs MD simulations in explicit POPC:Chol 2:1, POPC:Chol 2:1 at 310K, and DOPC:Chol 2:1 membrane environment were performed; (*ii*) for the CB_1_wt-ORG27569 system two 1 μs MD simulations, in POPC:Chol 2:1, starting from different binding conformations, were carried out; (*iii*) for the CB_1_-(H^2.41^L)-ORG27569 system, a 1 μs MD simulation long was performed in POPC:Chol 2:1.

Along all the trajectories, the CB_1_wt-ORG27569 simulations revealed the docking-derived binding mode being highly stable (See Supplementary Fig. S7). On the contrary, in the CB_1_-H^2.41^L dynamics, a substantial fluctuation of ORG27569 was appreciable, accordingly with mutagenesis data (See Supplementary Fig. S7). In the attempt to comprehend the CB_1_ structural changes upon allosteric ligand binding, a comparison between the CB_1_wt and the CB_1_wt-ORG27569 simulations was performed (Fig. 2b–e) and revealed that ORG27569 binding could cause an H-bond loss between the H^2.41^ Nε (TM2) and the R148 backbone oxygen (ICL1, Fig. 2b,c and Supplementary Fig. S8). Thus, by weakening the TM2/ICL1 interactions the allosteric ligand could allow a ICL1 rearrangement and eventually promote the formation of a salt bridge between R150 (ICL1) and D^8.49^ (H8) (Fig. 2a–d and Supplementary Fig. S8 and S9). This confirms previously published results showing H8 and ICL1 domains implicated in G-protein coupling or receptor internalization, in CB_1_^36^,^37^ as well as in other GPCR such as Rhodopsin^38^, or α_2A_-adrenergic receptor^39^. Noteworthy, these observations were statistically supported by all the MD simulations carried out in different conditions as above introduced (See Supplementary Fig. S8 and S9).

Upon ORG27569 binding, a pronounced displacement of the TM3 C-terminus region was observed at the T^3.46^ level (Fig. 2e and Supplementary Fig. S10), which has been implicated in the so called Hydrophobic Hindering Mechanism (HHM), in CB_1_ and other GPCRs^17^,^40^,^41^, supporting the idea that the alteration of this region, by the presence of ORG27569, may affect the orthosteric ligand binding affinity through the TM3 displacement.

Three-dimensional superposition of all the GPCR X-ray structures disclosed so far (See Supplementary Fig. S11) revealed that, the herein identified CB1 site (P2 site) corresponds to a CCM, a sequence shown to be sufficient to dictate cholesterol binding in many GPCRs^28^. When we analyzed the unbound CB_1_ dynamics in the three explicit membrane conditions (see Methods and Supplementary Fig. S12) several cholesterol molecules were found interacting with different domains of the CB_1_ receptor, as expected, (Fig. 2f and Supplementary Fig. S12). Interestingly one of them was actually found accommodated in the P2 cleft (Fig. 2g,h and Supplementary Fig. S12) adopting a binding conformation similar to that observed in the CCM pocket of other GPCR X-ray structures (See Supplementary Fig. S13)^28^.

The aforementioned observations suggested an intriguing scenario, for which change in cholesterol concentration and membrane composition could affect ORG27569 binding and even functionally compete with it.

### Functional competition between ORG27569 and cholesterol modulates CB_1_ affinity for inverse agonist

**Figure 3:**
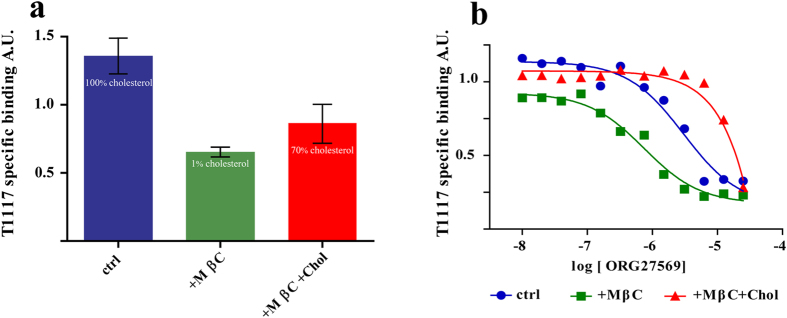
Functional competition between ORG27569 and cholesterol. (**a**) Rat brain membranes were left untreated (blue bar) or were cholesterol depleted by treatement with MβC (10 mM, 15 minutes) to be then replenished (red bar) or not (green bar) with soluble cholesterol (1 mM, 15 minutes). In each bar is indicated the amount cholesterol measured in the membranes after each treatment (expressed as % of the amount present in untreated samples, see methods for details). T1117 binding measurement performed as described in Fig.1b. Specific binding is indicated (data depict the mean +/− s.e.m. and are representative of 4 independent experiments. P < 0.05. One-way ANOVA-test was employed). (**b**) Rat brain membranes were treated with MβC and then replenished or not with cholesterol as described above. Membranes were incubated with the indicated amount of ORG27569. T1117 (2.5 μM) was then added and specific binding measured as described in Fig. 1b. Data were fitted with a dose response curve as described in the Method Sections. (data depict the mean +/− s.e.m. and are representative of three or more independent experiments. One-way ANOVA was employed. P < 0.05).

Cholesterol has been shown to affect GPCRs either directly, by binding to them and affecting their conformation, or indirectly, by influencing the membranous environment in which they are embedded. Effect of cholesterol and its precursor pregnenolone on CB_1_ binding was already demonstrated, with the lipids increasing the affinity of the receptor for inverse agonists^21^,^22^. We started proving that depletion of cholesterol, similarly to ORG27569, reduces CB_1_ affinity for T1117 (Fig. 3a,b). Rat brain membranes were treated or not with methyl-β-cyclodextrin (MβD) to selectively extract cholesterol and thus T1117 binding was measured. Treatment with MβD (98–99% of total cholesterol extracted) drastically reduced T1117 binding. The loss of affinity for T1117 is indeed due to cholesterol withdrawal since the exogenous replenishment of cholesterol (50–70% of total cholesterol re-uptake) recovered the ability to bind the inverse agonist (Fig. 3a). This suggests that, in absence of cholesterol, the conformation of CB_1_ is less prone to bind the inverse agonist T1117.

The IC_50_ of ORG27569 for CB_1_ was then measured in cholesterol depleted membranes. After MβD treatment or cholesterol replenishment, membranes were incubated with increasing concentration of ORG27569. T1117 specific binding and IC_50_ for ORG27569 were then measured (Fig. 3b). As already seen before (Fig. 3a), upon MβD treatment, the total amount of T1117 bound to the CB_1_ was reduced. Moreover, in the absence of cholesterol, ORG27569 shows a threefold lower IC_50_ if compared to that obtained with untreated membranes (900 nM and 3.1 μM, respectively). When MβD treated membranes were replenished with cholesterol, CB_1_ re-gained the ability to bind T1117, and ORG27569 IC_50_ rose in the high micromolar range (Fig. 3b). Thus, on rat brain membranes, cholesterol increases the binding of the inverse agonist (T1117) while decreases the IC_50_ of ORG27569.

### Functional competition between ORG27569 and cholesterol influences CB_1_ distribution at Plasma Membrane

**Figure 4:**
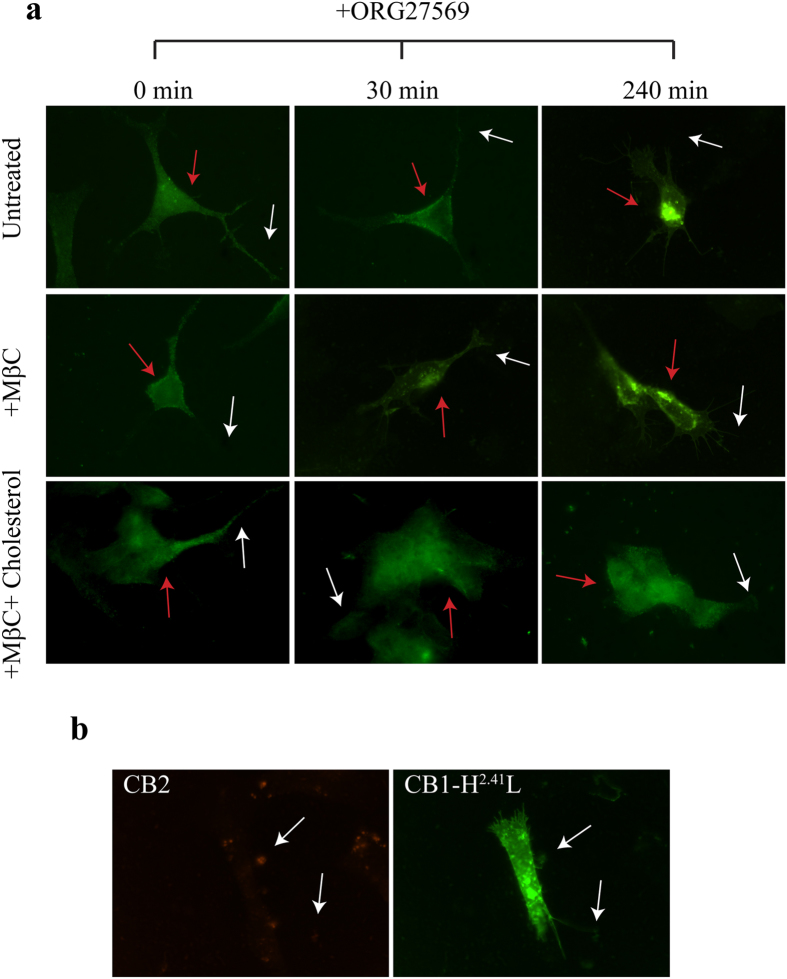
ORG27569 and cholesterol dependent shuttling of CB_1_ among axons and soma of the neurons. SHSY-5Y were treated with MβC and replenished or not with cholesterol. After cholesterol manipulation cells were treated or not with ORG27569 (3 μM) for the indicated time. After being fixed and permeabilized, cells were processed for immunfluorescence to detect endogenous CB_1_ receptor (panel (**a**)), endogenous CB_2_ (panel (**b**)) or transiently expressed CB_1_-H^2.41^L-GFP (panel (**b**)). White arrows and red arrows indicate axonal region and soma of the cell, respectively.

The effects of competition between ORG27569 and cholesterol were analyzed in cultured cells by following CB_1_ intracellular localization (Fig. 4). As already shown, upon agonist treatment, CB_1_ rapidly moves from the axons/dendrites to the neuron soma^42^ where endocytosis via chlatrin coated vesicles and receptor recycling occur. CB_1_ diffusion between axons/dendrites and soma was shown to be essential for its function^43^. We thus decided to follow change in CB_1_ localization in neurons upon ORG27569 treatment. After short treatment (30 minutes) with the allosteric molecule, the endogenous CB_1_ moved from axons to the cell soma (Fig. 4a), similarly to what has been reported after agonist treatment^42^. Noteworthy, longer treatment with ORG27569 (4 hours) induced the internalization of CB_1_. The effect of treatment with ORG27569 was specific for CB_1_ since neither CB_1_(H^2.41^L)-GFP or CB_2_ changed their localization after treatment with the allosteric molecule (Fig. 4b).

We thus subjected neuronal cells, treated with MβD and replenished or not with cholesterol, to short and long ORG27569 treatment. Similarly to ORG27569 treatment, when cholesterol was depleted by MβD (100% of cholesterol extracted), CB_1_ moved from dendrites to the central body of the neuron (Fig. 4a). This change in localization was reverted by cholesterol replenishment (70% of strarting cholesterol re-uptake), after which CB_1_ localization moved back to the dendrites. In the absence of cholesterol, the endocitosis induced by ORG27569 was accelerated being visible already after 30 minutes of incubation with the allosteric molecule (Fig. 4a). However enrichment of CB_1_ in the lysosome was somehow delayed compared to undepleted cells treated with ORG27569, probably for the effect, already postulated, that severe cholesterol depletion has on the endosomal-lysosomal route^34^. On contrary, in cell replenished with cholesterol incubation with ORG27569 did not induce CB_1_ internalization confirming that the two molecules compete (Fig. 4a) influencing the topological distribution of CB_1_ between two functionally different regions of neurons the axonal/dendrites part and the soma of the neurons.

## Discussion

A consensus pocket prediction on the entire CB_1_ receptor revealed nine potential allosteric sites. On the basis that ORG27569 selectively binds CB_1_ over CB_2_^23^,^24^, and through mutagenesis experiments, we identified P2 as a ORG27569 binding site. **ORG27569alk3**, a derivatized version of the allosteric ligand, physically interacted with P2 addressing S^2.45^ or S^3.42^ (Fig. 1). *In silico* simulations were performed to reveal the ORG27569 binding mode and the CB_1_ structural changes upon allosteric binding. The simulations strongly suggested that the ORG27569 binding elicits a TM3 displacement. This could be one of the major factor affecting the orthosteric agonist CP55940 binding affinity, in line with the observation that T^3.46^I mutation in CB_1_ as well as mutations on TM3, such as the L^3.29^I and A^3.34^M mutants here made, do affect the orthosteric site. In addition, upon binding of ORG27569, a H8-ICL1 rearrangement occurs (See Supplementary Fig. S8 and S9), and accordingly to already published experimental data^36^,^37^, this could explain the ORG27569 effect in blocking the CB_1_ coupling to its cognate G-protein.

A detailed analysis of the localization of allosteric pockets in other Class A GPCRs revealed that CB_1_ P2 site corresponds to a CCM, a motif found in 26% of class A GPCRs^28^. However, in a previous study, recently reported by Stevens at *al.*,^28^ CB_1_ was not included in the list of receptors possessing the CCM. Although all the interactions between cholesterol and P2 are conserved with respect to those detected with others CCM regions, the primary amino acid sequence of CB_1_ P2 site does not completely fulfill the CCM consensus requirements and thus has not been detected. Not far from this cholesterol binding region (P2 pocket) and located in the bottom part of the 7 TM-bundle, another cholesterol binding pocket exists (CRAC, Cholesterol Recognition Amino Acid Consensus sequence, L/V-(X)_1–5_-Y-(X)_1–5_-R/K)^22^ and corresponds to P1 pocket in our model (Fig. 1a). CRAC and CCM are related by inversion. The existence of multiple cholesterol sites was somehow predictable since mutations in the CRAC region do not affect CB_1_ localization^22^.

Functional competition between cholesterol and ORG27569 was here demonstrated acting at least on two levels: i) as shown by functional competition assays (Figs 3,4), the binding of the two molecules oppositely influences affinity of CB_1_ for the inverse agonist AM251; ii) as shown following CB_1_ intracellular localization, cholesterol and ORG27569 treatments compartmentalize the receptor to the axon and to the soma of the neuron, respectively (Figs 3,4). All together, these results enlighten a scenario where cholesterol, an endogenous negative modulator of CB_1_, and exogenous allosteric molecules compete for imposing specific CB_1_ conformations and affect its shuttling between functionally different regions of the neurons. In resting neurons, CB_1_ is localized in lipid rafts at the axons of the cells. Noteworthy, these rafts contain the entire endocannabinoid machinery^44^,^45^. In fact, besides cholesterol, lipid rafts are also enriched in *i*) beta2–arrestin *ii*) G-proteins, *iii*) anandamide^45^,^46^,^47^. Accordingly, *in vitro* experiments demonstrated that G-protein coupling happens at the lipid rafts^48^,^49^. As expected, agonist binding to CB_1_ induces a receptor conformation change that activates G-proteins and moves the receptor to the soma, where it gets internalized^48^,^49^. Surprisingly, the same internalization path induced by the agonist happens upon ORG27569 binding. Being able to control the lateral diffusion of the receptor, ORG27569 takes away CB_1_ from its endogenous regulation, controlling its function^44^.

Despite our computational analysis points to explain the experimentally observed functional competition between ORG27569 and cholesterol with them competing for the same site, we cannot firmly exclude this not being the case for CB_1_. Indeed, it is equally plausible that cholesterol could bind to the receptor at sites different from P2 and that it rather influences CB_1_ sensitivity to ORG27569 by changing the overall conformation of the receptor or affecting non-specific lipid-protein interactions. Noteworthy, these different effects of cholesterol are not mutual excluding and they can be all existing and participating to the mechanism underpinning functional competition between the two allosteric molecules. In favour of the existence of Cholesterol/ORG27569 competition for the same CCM argues the effect of the F^8.54^A mutation (CRAC, H8, Fig. 1a), that, in our hands, generates a receptor more sensitive to ORG27569. Indeed, mutation of a bulky aromatic residue (Phe) with a small lipophilic one (Ala) and/or the displacement of a bound cholesterol molecule possible consequence of such mutation, would facilitate conformational changes induced by ORG27569. By imposing TM2-4 packing, cholesterol would have an opposite effect with respect to ORG27569, which drifts these helices apart (Fig. 2c–h).

Although many conformational, pharmacological and signaling features of GPCRs have been extensively studied, many aspects related to their interaction with membrane lipids are just beginning to be addressed. The knowledge that more than one site for cholesterol binding exist, on one hand, and the discovery of the exact locations of those pockets, on the other, will surely help to better characterize the precise mechanism of cholesterol modulation in GPCRs, which still remains partially hidden. Herein we demonstrated that P2 is druggable, surely in CB_1_ and likely in others GPCRs, by exogenous ligands albeit structurally unrelated to cholesterol. Indeed, if cholesterol would target the same binding ORG27569 site, the two molecules would address the pocket in a highly different way. This finding suggests that the CCM sites of any GPCRs can be in principle targeted to obtain strong, selective, novel, allosteric modulators depending on the conservation among different GPCRs of the residues engaged. ORG27569 is an amazing example being able to distinguish even between highly related CB_1_ and CB_2_ receptors. The deep comprehension of the lipid effect/roles on 7TM bundle receptors surely represents one of the major challenges we have still to face in the GPCRs field. Efforts in this direction would enhance significantly our ability to design efficacious, useful and probably safer therapeutic agents.

## Methods

### Reagents

Salt and Organic solvents were from Sigma Aldrich (U.S.A.), Applichem (Germany) and Carlo Erba (Italy). FITC and Texas Red coniugated monoclonal and polyclonal secondary antibodies were from Sigma Aldrich (U.S.A). T1117 (Tocrifluor) (*N*-(Piperidin-1-yl)-5-(4-(4-(3-(5-carboxamido-tetramethylrhodaminyl)-propyl))phenyl)-1-(2,4-dichlorophenyl)-4-methyl-1*H*-pyrazole-3-carboxamide), AM251 (*N*-(Piperidin-1-yl)-5-(4-iodophenyl)-1-(2,4-dichlorophenyl)-4-methyl-1*H*-pyrazole-3-carboxamide) were from TOCRIS Bioscience. T1117 and AM251 were reconstituted in EtOH and diluted in PBS to 0.010 mM and 1.08 mM, respectively. ORG27569 (5-Chloro-3-ethyl-*N*-[2-[4-(1-piperidinyl)phenyl]ethyl-1*H*-indole-2-carboxamide) was reconstituted 10 mM in DMSO. PBS tablets were from Fluka.

### Cell Cultures and DNA transfection

HuH7, HEK293 cells were cultured in DMEM supplemented with 10% FBS. SHSY-5Y^50^ were cultured in DMEM/F12 medium supplemented with 10% FBS and non essential amminoacids. All cultures were grown in an atmosphere of 5% CO_2_ at 37 °C. Freshly defrost cells were used for the transfection experiments. After a maximum of 7 days in culture cell were splitted the day before the experiment to gain a plate at 20–30% confluence. Poliethylenimmine (PEI) in water (1 μg/μl) was used as transfecting agent. Briefly 4 μg of DNA were mixed with 10 μg of PEI in 150 mM NaCl to be then added after 30 minutes of incubation to a 10 cm dish of cells in complete fresh medium.

### Mutagenesis

cDNA coding for the full length human CB_1_ (NM_016083) or rat 3xFLAG-CB_1_-GFP (kindly provided by professor Zsolt Lenkei) cloned in pcDNA3.1 (Invitrogen) was used as template for the PCR mutagenesis. 2 Units PFU Polymerase (Promega) was supplemented with 50 ng of template cDNA, 125 ng of primers, 200 μM of each dNTP according manufacturer instructions. After 1 minutes of denaturation at 95 °C , 18 PCR cycles (30 seconds at 95 °C, 1 minutes at 55 °C, 10 minutes at 68 °C) were performed. After the reaction, samples were digested with the restriction enzyme DpnI (BioLabs) for 1 hour at 37 °C and transformed in DH5α competent cells. The sequence of the mutants were confirmed by sequencing of the DNA. The Upstream (up) and downstream (dw) primers used to introduce the indicated single amminoacidic sustituitions are listed in Supplementary Methods.

### Membrane preparation from cultured cells

Cells were harvested 48 hours after the transfection and centrifuged for 5 minutes at 800 × g, resuspended in cold PBS, and repelleted again. Cell pellet were dounced 20 times in a Teflon dounce. Homogenates were centrifuged for 5 minutes at 1,000 × g (4 °C) to remove nuclei, cell debris and unbroken cells. The resulting was centrifuged at 20,000 × g to obtain a membrane fraction used for the fluorescence experiments.

### Membranes preparation from Rat Brain

Adult (300–400 g), male Sprague-Dawley rats (kindly provided by Prof. Sorrentino and Prof. Ialenti, Faculty of Pharmacy, Naples, Italy) were killed by decapitation. The brains were rapidly removed and chilled in ice-cold PBS. Each organ was disrupted in 20 ml of cold PBS using a Teflon dounce (20 passages). The homogenates were centrifuged at 1,000 × g (4 °C) for 30 minutes to remove cell debris and unbroken tissues. The supernatant was centrifuged at 20,000 × g to and the resulting pellet frozen on solid CO_2_.

### ORG27569 treatment

Membranes Homogenates were incubated with the indicated concentration of ORG27569 before being processed for fluorescence binding measurement. Cell in culture were incubated with 3 μM ORG27569 for the indicated amount of time to be then fixed and processed for immunofluorescence as described below.

### MβD treatment and cholesterol replenishment

Membrane Homogenates were incubated with 10 mM MβD (Sigma) for 15 minutes before being processed for fluorescence binding measurement. When indicated soluble cholesterol (1 mM) was added to the membranes for further 15 minutes. Cultured Cells were incubated with 10 mM MβD (Sigma) for 15 minutes dissolved in PBS 0,1% BSA. When indicated soluble cholesterol (0,5–1 mM dissolved by sonication in PBS 0,1% BSA) was added to the cell after MβD. To determine the rate of cholesterol depletion or addition, we measured cellular cholesterol levels by a colorimetric assay (cholesterol/cholesteryl ester quantification; Calbiochem, La Jolla, CA) according to the manufacturer’s instructions. Following cholesterol addition, membranes did re-uptake amounts of cholesterol ranging from 50 to 70% of total cellular cholesterol.

### T1117 Fluorescent measurement

Binding to T1117 was measured as previously described (Bruno *et al.*, 2014)^32^. See Supplementary Methods for details.

### Immunofluorescence

HuH7 and SHSY-5Y growing on glass coverslips were fixed in 4% Formaldehyde dissolved in PBS for 30 minutes. Formaldehyde was quenched by incubating the coverslips for 30 minutes in 0,1M Glycine dissolved in PBS. Cells were permeabilized in 0,1% TritonX100 for 10 minutes at RT to be then incubated with primary and secondary antibody diluted in PBS for 1 hour and 30 minutes, respectively. In order to measure the ratio between levels of PM and intracellular 3xFLAG-CB_1_-GFP protein forms, cells were incubated after fixation without permeabilization with a rabbit polyclonal anti-FLAG antibody followed by a Texas-Red coniugated secondary antibody. The immunofluorescence intensity in the Texas-Red channel (depending only on the PM localized CB_1_) was measured using NIH ImageJ Biophotonic programs and normalized to one of the GFP channel (depending on the total CB_1_-GFP expression, PM + intracellular). For each transfection, 20 cells were considered for quantification. The results are given as mean +/− s.d.m. The following dilutions were used: polyclonal antiCB_1_ (Santa Cruz) 1:50, polyclonal antiCB_2_ 1:50 (Santa Cruz), Texas-Red anti-rabbit (Sigma) 1:400. Immunfluorescence images were taken by a Leica DFC320 video-camera (Leica, Milan, Italy) connected to a Leica DMRB microscope equipped with a 100 X objective and the Image J Software (National Institutes of Health, Bethesda, MD) was used for analysis.

### CB_1_ immunoisolation from cultured cells and Proteinase K digestion

Cells transiently expressing rat CB_1_-GFP were lysed in B-Buffer (Hepes K-OH 50 mM, 150 mM NaCl, 1% Tryton X-100 supplemented with Protease Inhibitors). Lysates were centrifuged at 14.000 rpm to remove cell debris and unbroken cells. Clarified lysates were incubated with the primary antibody (over night, 4 °C) followed by Protein-A coupled Sepharose (45 min 4 °C). Samples were extensively washed in B-Buffer to be then run on SDS-PAGE. Samples were in gel digested with Proteinase K (100 ng/μl in 50 mM Hepes buffer, pH 7.8 supplementaed with 1 mM CaCl2). 20 μl of the digestion were processed for LC/MS.

### HPLC/MS

All samples were analyzed by analytical HPLC/MS (Agilent 1200 series HPLC system, Agilent 1260 UV-Vis detector Infinity and Agilent Quadrupole 6110 LC/MS) equipped with a C18-bounded analytical reverse-phase HPLC column (Vydac 218TP104, 4.6 × 250 mm) using a gradient elution (10 to 90% acetonitrile in water (0.1% TFA) over 20 min; flow rate = 1.0 mL/min.

### LC/MS Spectra Analysis

LC/MS spectra were analyzed with MetAlign with the following setting [Mass resolution/BIN (nominal mass mode, 0,65), Peak slope factor (5 × noise), Peak Threshold factor (5 × noise), Peak Threshold Abs value (150), Average peak width (3 scans), Autoscaling on total signal, Tuning alignment (preAlign Processing Iterative, Mass peak selection set on Min Factor (5 × noise)]. Amplitudes of masses coming from treated and untreated samples were compared to identify mass exclusively present in each of the sample. Masses were assigned with Mascot (MatrixScience). Samples containing either Proteinase K, Protein Sepharose or antibody were run as control.

### Computational Protocol

#### Homology model

The CB_1_ model was built as previously reported by us^32^, (Supplementary Table 1 and 2 and Supplementary Fig. S1 and S2). For details see Supplemental Materials.

#### Generation of the initial CB_1_-membrane complex and MD simulations setting

Experimental evidences support that palmitoylation at position C415 is fundamental for proper CB_1_ functionality^36^. Therefore, the refined CB_1_ model was palmitoylated at position C415, and the first *N*-terminal (S87) and the last *C*-terminal (E416) residues were capped with ACE and NME respectively. The generated model was embedded in an explicit POPC/Cholesterol (2:1) bilayer, applying a protocol earlier described^51^.

Molecular dynamics (MD) simulations were performed using NAMD2.9 software and using the *Amber99SBildn* and *lipid11* as force field, atom type and parameters for the palmitoyl molecule were retrieved from *lipid11* and *gaff* force field (for the acyl chain, the carbonyl group and for the thioester bond, respectively). For details see Supplementary Informations.

#### Consensus Pocket Prediction and Mutant Selections

The refined 3D model and the relaxed structure (after 50 ns of MD simulations) of the CB_1_ receptor were used to identify the ORG27569 binding site. We decided to employ three well-established algorithms: (*i*) FTMAP^52^, (*ii*) PocketFinder^53^, and (*iii*) Q-SiteFinder^54^. For each algorithm 10 druggable pockets were considered (Supplementary Fig. S3 and S4). Mutants were selected according to the following criteria: (*i*) since ORG27569 selectively bind CB_1_^23^,^24^ only the pockets showing great sequence diversity with respect to CB_2_ sequence were take into account (Supplementary Fig. S4); (*ii*) when possible (CB_1_-CB_2%_ ID = 42.50) only non conservative mutation were considered (i.e. His to Leu); (*iii*) in the attempt to avoid mutation leading to non-functional mutant receptors CB_1_ amino acids were mutated in the corresponding CB_2_ residues and not in Ala (Supplementary Fig. S4).

#### Docking Studies

The refined CB_1_ receptor (50 ns) was used to carry out docking studies. ORG27569 was built using the fragment builder tool of Maestro9.1 (See Supplementary Methods for details).

#### MD Simulations of CB_1_wt, CB_1_wt-ORG27569 bound and CB_1_-(H^2.41^L)-ORG27569 systems

MD simulations of the CB_1_wt, CB_1_wt-ORG27569 and CB_1_-(H^2.41^L)-ORG27569 complexes were conducted as previously described for 1 μs for each system (For details see Supplementary Methods section).

## Additional Information

**How to cite this article**: Stornaiuolo, M. *et al.* Endogenous vs Exogenous Allosteric Modulators in GPCRs: A dispute for shuttling CB_1_ among different membrane microenvironments. *Sci. Rep.* **5**, 15453; doi: 10.1038/srep15453 (2015).

## Supplementary Material

Supplementary Information

## Figures and Tables

**Table 1:** List of the generated CB_1_ mutants.

Mutation List				
Pocket	Residue Exchange			
	Position	CB_1_	CB_2_	Mutation
P1	1.45	Phe	Leu	F^1.45^L
	7.51	Ile	Val	I^7.51^V
	8.54	Phe	Ala	F^8.54^A
P2	1.55	Cys	Tyr	C^1.55^Y
	2.41	His	Leu	H^2.41^L
	4.46	Phe	Leu	F^4.46^L
P3	3.27	Phe	Leu	F^3.27^L
	3.34	Ala	Met	A^3.34^M
	4.54	Ile	Ala	I^4.54^A
P4	3.29	Leu	Ile	L^3.29^I
	6.58	Asp	Ser	D^6.58^S
	7.33	Thr	Lys	T^7.33^K
P5	4.52	Ile	Leu	I^4.52^L
	4.56	Ile	Val	I^4.56^V
	5.46	Val	Phe	V^5.46^F
