# Supplementary material for: Endogenous vs Exogenous Allosteric Modulators in GPCRs: A dispute for shuttling CB1 among different membrane microenvironments
Source: Sci Rep. 2015 Oct 20;5:15453. doi: 10.1038/srep15453 (PMC4612305; doi:10.1038/srep15453)
Supplement: Supplementary Information [file srep15453-s1.pdf]

Supplementary Information

Endogenous vs Exogenous Allosteric Modulators in GPCRs:

A Dispute for shuttling CB1 among different membrane microenviroments

Mariano Stornaiuolo,[1,#] Agostino Bruno,[1,#] Lorenzo Botta,[1] Giuseppe La Regina,[2] Sandro Cosconati,[3] Romano Silvestri,[2] Luciana Marinelli [1] & Ettore Novellino.[1]

[1]Department of Pharmacy, University of Naples ‘‘Federico II’’, via D. Montesano 49, 80131 Naples, Italy. [2]Istituto Pasteur–Fondazione Cenci Bolognetti, Dipartimento di Chimica e Tecnologie del Farmaco, Sapienza Università di Roma, Piazzale Aldo Moro 5, I-00185 Roma, Italy. [3]DiSTABiF, Seconda Università di Napoli, Via Vivaldi 43, 81100 Caserta, Italy

[#] These authors contributed equally to this work

Corresponding authors: Luciana Marinelli: lmarinelli@unina.it

Mariano Stornaiuolo: mariano.stornaiuolo@gmail.com

SUPPLEMENTARY RESULTS

1. HOMOLOGY MODELS

Supplementary Table 1 | Identity Matrix

Supplementary Table 2. | TM Identity Matrix

Supplementary Figure 1 | $S1P_1$-CB_1 sequences alignment

Supplementary Figure 2 | Refined CB_1 homology models

2. GENERATION OF THE CB_1WT-MEMBRANE COMPLEX AND MD SIMULATIONS SETTING

Supplementary Table 3 | MD setting

3. CONSENSUS POCKET PREDICTION AND MUTANTS SELECTION

Supplementary Figure 3 | Consensus Pocket Prediction

Supplementary Figure 4 | Mutant Selection

4. LIQUID CHROMATOGRAPHY/MASS SPECTROMETRY (LC/MS) DATA

Supplementary Figure 5 | LC/MS analysis of ORG27569alkF3 treated CB_1

Supplementary Figure 6 | Measurement of PM localization of CB1

5. BINDING MODE RELIABILITY AND ANALYSIS OF THE TRAJECTORIES

Supplementary Figure 7 | Statistical analysis of the binding mode stability

Supplementary Figure 8 | Statistical analysis of the structural effects induced by ORG27569

Supplementary Figure 9 | Conformational Analysis of ICL1-H8 in the different simulations

Supplementary Figure 10| Statistical analysis of the TM3 effects induced by ORG27569

6. ANALYSIS OF GPCR X-RAY CRYSTAL STRUCTURES IN COMPLEX WITH CHOLESTEROL, AND CHOLESTEROL INTERACTION WITH CB_1WT

Supplementary Figure 11 | X-ray crystal structures analysis

Supplementary Figure 12 | Analysis of cholesterol binding in different membrane environment

Supplementary Figure 13 | Cholesterol binding conformations

7. GENERATION OF THE $CB_1(H^{2.41}L)$-ORG27569 SYSTEM

Supplementary Figure 14 | Staring Conformations

Supplementary Table 4 | MD simulations carried out.

8. APPENDIX A

Structure Selection

Supplementary Figure 15 | Calculations of the Cholesterol Probability Distribution

9. CHEMISTRY SECTION

SUPPLEMENTARY METHODS

Mutagenesis

T1117 Fluorescent measurement.

Docking studies

MD Simulations of CB1 unbound, CB1wt-ORG27569 bound and CB1-($H^{2.41}L$)-ORG27569 bound systems

SUPPLEMENTARY REFERENCES

SUPPLEMENTARY RESULTS

1. Homology models

To date, 400 class A GPCRs are known as potential drug target[1]. However, only for 23 of them the structure was solved by X-ray crystallographic studies. In this regards, for structure-based purpose, aimed at discovering new GPCR ligands as drugs, the homology modeling approach is still essential. Several models of the CB_1 receptor have been proposed until now using rhodopsin, β_2-adrenergic and adenosine receptor subtype 2A (A_{2A}) as template[2–4]. Among the possible CB_1 templates, recently, the human sphingosine 1-phosphate receptor ($S1P_1$) has been disclosed offering new possibility to build up more reliable 3D model for the CB_1 receptor[5,6]. $S1P_1$ TM helices shows an average sequence identity of about 35%, with respect to CB_1 helices (Supplementary Table 1 and 2), making it a suitable template to build a 3D model of CB_1. Receptors having the highest sequence identity with respect to the CB_1, if the entire sequence are considered, are the $S1P_1$ and the A_{2A}{27% and 23% of sequence identity, respectively [(data obtained from the ClustalW identity matrix)[7] see Supplementary Table 1 and 2]}. Moreover, $S1P_1$ orthosteric binding site was evolutionary selected to bind sphingosine (a lipid-derived ligand) and similarly CB_1 binds a lipid-derived ligand (anandamide) as transmitter[8,9]. In addition, experimental evidences support the notion that CB_1 and $S1P_1$ share a common mechanism of binding, a common activation mechanism, and that sphingosine and its analogs interact with the CB_1 receptor[6,9]. Therefore, the $S1P_1$ X-ray crystal structure (pdb code: 3V2Y) was choose and used as template to generate the 3D structure of CB_1. CB_1 and $S1P_1$ sequences were aligned using the ClustalW server[7] (Supplementary Fig. 1) and the 3D model of CB_1 was generated using the Modeller9.11 software[10].

Supplementary Table 1 | Identity Matrix. Percent Identity Matrix obtained from Clustal2.1.

	P21554 CNR1 HUMAN	P21453 S1PR1 HUMAN	P29274 AA2AR HUMAN	P07550 ADRB2 HUMAN	P02699 OPSD BOVIN
**P21554 CNR1 HUMAN**	**100**	**27,03**	**23,41**	18,37	17,35
**P21453 S1PR1 HUMAN**	27,03	**100**	25,29	18,33	17,77
**P29274 AA2AR HUMAN**	23,41	25,29	**100**	26,3	18,47
**P07550 ADRB2 HUMAN**	18,37	18,33	26,3	**100**	16,96
**P02699 OPSD BOVIN**	17,35	17,77	18,47	16,96	**100**

Supplementary Table 2. | TM Identity Matrix. Identity percentage for relevant CB_1 and $S1P_1$ domains.

Domain	% ID
TM1	24.24
TM2	42.31
TM3	34.38
TM4	33.33
TM5	35.48
TM6	34.29
TM7-H8	39.47
ECL2	40
**Average**	**35.43**

Supplementary Figure 1 | $S1P_1$-CB_1 sequences alignment. Sequence alignment between CB_1 and $S1P_1$ receptors. Highlighted in light grey residues included in TM domains, in red the cysteine residues forming disulphide bond, while in green and bold conserved residues in Class A GPCR. In yellow the initial and final residues considered to build the CB_1 model. Finally in cyan the IL3 portion substituted with the TL4 lysozyme in the X-ray structure of the $S1P_1$ receptor.

```
CNR1_HUMAN       MKSILDGLADTTFRTITTDLLYVGSNDIQYEDIKGDMASKLGYFPQKFPL 50
S1PR1_HUMAN      ----------------------------------------MG--PTSVPL 8

CNR1_HUMAN       TSFRGSPFQEKMTAGDNPQLVPADQVNITEFYNKSLSSFKENEENIQCGE 100
S1PR1_HUMAN      VKAHRS-----------------------------SVSDYVNYDIIVRHY 49

CNR1_HUMAN       NFMDIECFMVLNPSQQLAIAVLSLTLGTFTVLENLLVLCVILHSRSLRCR 150
S1PR1_HUMAN      NYTGKLNISADKENSIKLTSVVFILICCFIILENIFVLLTIWKTKKF-HR 78

CNR1_HUMAN       PSYHFIGSLAVADLLGSVIFVYSFIDFHVFHRKDSRNVFLFKLGGVTASF 200
S1PR1_HUMAN      PMYYFIGNLALSDLLAGVAYTANLLLSGATTYKLTPAQWFLREGSMFVAL 128

CNR1_HUMAN       TASVGSLFLTAIDRYISIHRPLAYKRIVTRPKAVVAFCLMWTIAIVIAVL 250
S1PR1_HUMAN      SASVFSLLAIAIERYITMLK--------NNFRLFLLISACWVISLILGGL 177

CNR1_HUMAN       PLLGWNCEKLQSVCSDIFPHIDETYLMFWIGVTSVLLLFIVYAYMYILWK 300
S1PR1_HUMAN      PIMGWNCISALSSCSTVLPLYHKHYILFCTTVFTLLLLSIVILYCRIYSL 227

CNR1_HUMAN       AHSHAVRMIQRGTQKSIIIHTSEDGKVQVTRPDQARMDIRLAKTLVLILV 350
S1PR1_HUMAN      VRT------------------------------RSSENVALLKTVIIVLS 263

CNR1_HUMAN       VLIICWGPLLAIMVYDVFGKMN-KLIKTVFAFCSMLCLLNSTVNPIIYAL 399
S1PR1_HUMAN      VFIACWAPLFILLLLDVGCKVKTCDILFRAEYFLVLAVLNSGTNPIIYTL 313

CNR1_HUMAN       RSKDLRHAFRSMFPSCEGTAQPLDNSMGDSDCLHKHANNAASVHRAAESC 449
S1PR1_HUMAN      TNKEMRRAFIRIMSCCKCPSGDSAGKFKRPIIAGMEFSRSKSDNSSH--- 360
```

```
CNR1_HUMAN        IKSTVKIAKVTMSVSTDTSAEAL 472
S1PR1_HUMAN       PQKDEGDNPETIMSSGNVNSSS- 382
```

Twenty 3D models of CB_1 were generated using Modeller9.11 software[10]. The obtained models were scored using the available scoring functions in Modeller9.11 software[10] (DOPE score), and through visual inspections. The best model had the conserved disulfide bond between C257 and C264 on extracellular loop 2 (ECL2). The protonation state of ionizable residues was fixed by using the Protein Preparation Wizard of the Maestro 9.1 graphical user interface,[11] and then checked by visual inspection. In this case the $E^{1.49}$ and $D^{2.50}$ residues were protonated, according to previous works.[12]

The geometry of the final model was optimized using several cycles of OPLS2005 force field (available in Maestro[11]) minimization, until a gradient of 0.001 kcal/mol*$Å^2$ was reached.

The loop of the optimized 3D model of the CB_1 receptor were preliminary refined using the refinement loop tool available in Modeller 9.11 software[10], and also in this case the obtained loop models were scored using the available scoring functions in Modeller9.11 software[10], and through visual inspections. The final model was embedded in an explicit POPC/Cholesterol (2:1) bilayer and refined through 50ns of MD simulations (further details about the generation of the membrane-receptor complex, and MD simulation setting can be found in the next sections of the Supplementary Information). The resulting model is reported in Supplementary Figure 2, where it can be appreciate that the F268 residue on ECL2 is nicely accommodate inside the CB_1 binding pocket, according to the experimental evidences that this residue is relevant for ligand binding.[13]

Supplementary Figure 2 | Refined CB_1 homology models.3D structure of the CB_1 homology model. Highlighted as surface and in blue the putative orthosteric binding pocket, while in yellow and spheres the F268 on ECL2 implicated in ligand binding.[13] Finally, in stick and blue the cysteine residues forming the disulphide bond on ECL2.

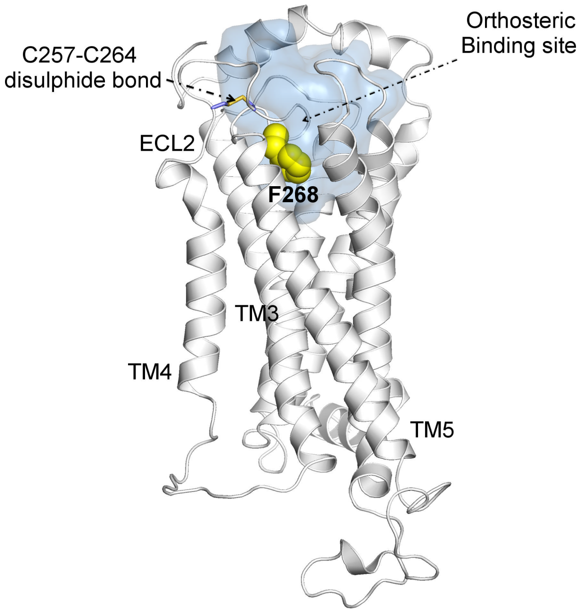

2. Generation of the CB_1wt-membrane complex and MD simulations setting

A pre-equilibrated POPC phospholipids bilayers was generated using the membrane-builder tool of charm-gui.org (http://www.charmm-gui.org) 94x94 Å with a ratio POPC:cholesterol equal to 2:1. In order to place the receptor into the bilayer a hole was generated, and lipids in close contact (<1 Å distance from any protein atoms) were deleted. In this case POPC or cholesterols molecules were manually deleted in each layer in order to retain the same cholesterol concentration ratio. The membrane-receptor complexes thus obtained were solvated and neutralized using the solvation and autoionize modules of VMD1.8.9[14,15] . The ionic strength was kept at 0.15 M by NaCl and we used TIP3 water model. Finally, the whole system was parametrized using the tleap tool of Amber12, and *Amber99SBildn*, *lipid11* and *gaff* as force field[16].

The CB_1-C415-palmitoyl/membrane complex was submitted to a relaxation protocol based on six steps and a preliminary production phase of 50ns (Supplementary Table 3). The production phases was performed using the NPT ensemble and Langevin as thermostat. The cutoff for the non-bonded interactions was set equal to 10 Å and a smooth switching function was used to truncate the van der Waals potential energy smoothly at the cutoff distance; the parameters that specifies the distance at which the switching function should start was set to 8 Å, with a pairlistdist of 12 Å, updated every 2 steps. The PME grid spacing was set with a resolution of 1 Å and an interpolation order of 4. The time step was set equal to 2 fs and the RATTLE algorithm was applied. The initial periodic

boundary conditions were set equal to 94.5 x 94.5 x 110.5 Å, and frames were written every 50.000 steps.

Supplementary Table 3 | MD setting. MD parameters setting.

				**Relaxation Phase**				
	**Phase**	**Steps/Time**	**Temp (K)**	**Harmonic Constraints kcal/mol*Å^2**				**Ensemble**
				Backbone	Sidechains	Lipid	Solvent	
Step1	Mini	3000	0	50	50	25	25	NVT
Step2	Mini	3000	0	25	12.5	-	-	NVT
Step3	Mini	3000	0	10	5	-	-	NVT
Step4	Heat/Pressure Equilibration	600ps	0→300	10 (Cα only)	5	-	-	NVT/NPT Switch every 10ps
Step5	Equil1	500ps	300	5 (Cα only)	1	-	-	NPT
Step6	Equil2	10ns	300	-	-	-	-	NPT
				**Production Phase**				
Step1	Production	50 ns	300	-	-	-	-	NPT

3. Consensus Pocket Prediction and Mutants Selection.

Reported in Supplementary Figure 3 the comparison of the 9 pockets identified by FTMAP[17,18], PocketFinder[19], and Q-SiteFinder[20] in the refined homology model and in the relaxed structure of the CB_1 receptor model. In Supplementary Figure 4 the sequence alignment between CB_1 and CB_2 receptor, and highlighted the selected single point mutations.

Supplementary Figure 3 | Consensus Pocket Prediction. Comparison of the probes localization for each software used (yellow=FTMAP, green QSiteFinder, and red=PocketFinder), and for two different conformation, the refined homology model (HM), and after 50ns of MD simulations (50ns).

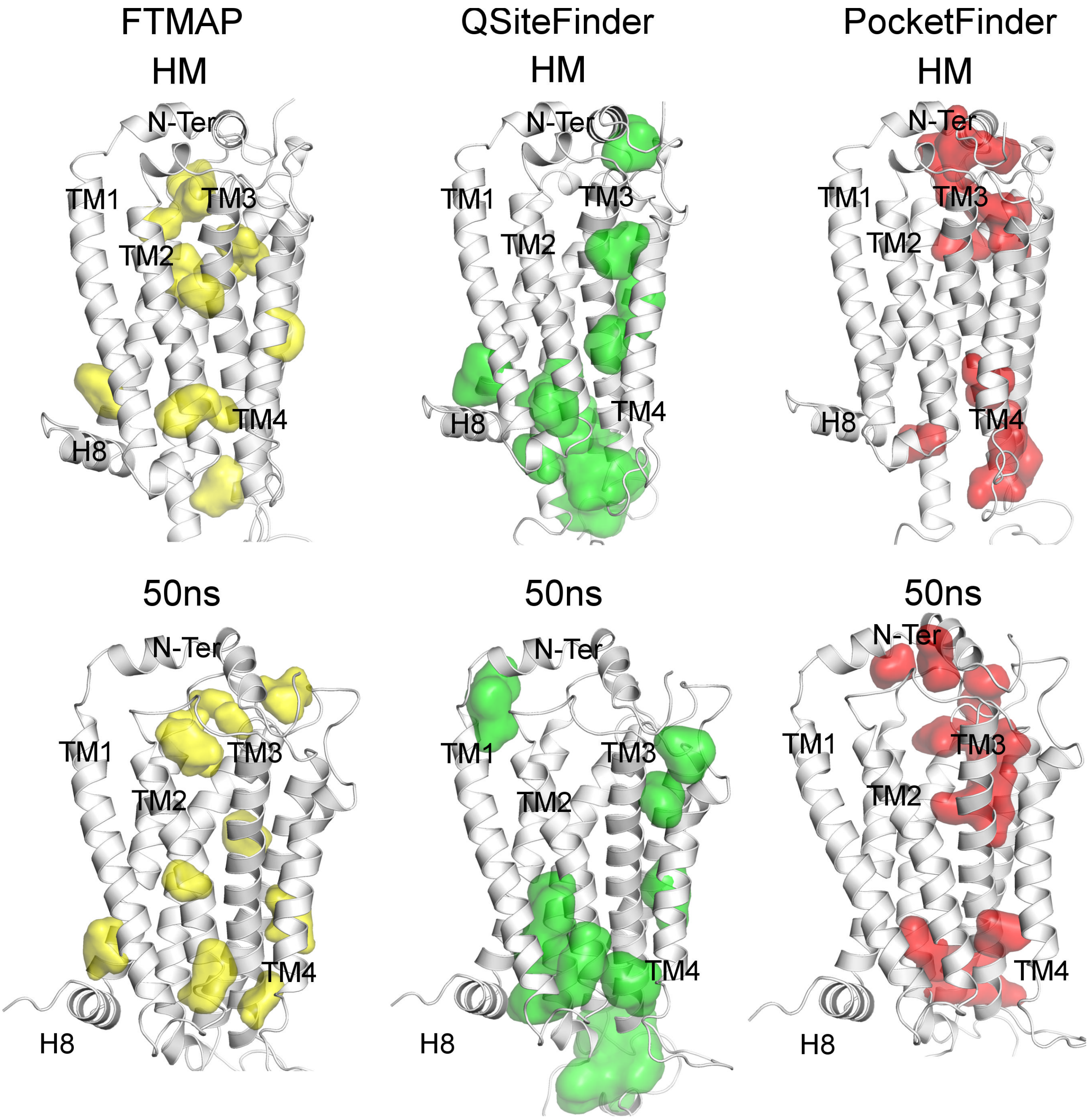

Supplementary Figure 4 | Mutant Selection. Sequence alignment between CB_1 and CB_2 receptors. Highlighted in gray the TM domains, in bolt and green the conserved residues of each TM domains for Class A GPCR, in bolt and red the cysteine residues forming the disulphide bond. Highlighted in green, cyan, yellow, blue and purple the sites where residue were mutated (According to the color code of Figure 1 Main Text).

```
sp|P21554|CNR1_HUMAN      MKSILDGLADTTFRTITTDLLYVGSNDIQYEDIKGDMASKLGYFPQKFPLTSFRGSPFQE 60
sp|P34972|CNR2_HUMAN      ------------------------------------------------------------

                                                                                TM1
sp|P21554|CNR1_HUMAN      KMTAGDNPQLVPADQVNITEFYNKSLSSFKENEENIQCGENFMDIECFMVLNPSQQLAIA 120
sp|P34972|CNR2_HUMAN      ------------MEECWVTEIANGSKDGLDSNP-----------MKDYMILSGPQKTAVA 37

                                                    I1              TM2
sp|P21554|CNR1_HUMAN      VLSLTLGTFTVLENLLVLCVILHSRSLRCRPSYHFIGSLAVADLLGSVIFVYSFIDFHVF 180
sp|P34972|CNR2_HUMAN      VLCTLLGLLSALENVAVLYLILSSHQLRRKPSYLFIGSLAGADFLASVVFACSFVNFHVF 97

                            E1                TM3                    I2          TM4
sp|P21554|CNR1_HUMAN      HRKDSRNVFLFKLGGVTASFTASVGSLFLTAIDRYISIHRPLAYKRIVTRPKAVVAFCLM 240
sp|P34972|CNR2_HUMAN      HGVDSKAVFLLKIGSVTMTFTASVGSLLLTAIDRYLCLRYPPSYKALLTRGRALVTLGIM 157

                               TM4                E2                       TM5
sp|P21554|CNR1_HUMAN      WTIAIVIAVLPLLGWNCEKLQSVCSDIFPHIDETYLMFWIGVTSVLLLFIVYAYMYILWK 300
sp|P34972|CNR2_HUMAN      WVLSALVSYLPLMGWTCCPRP--CSELFPLIPNDYLLSWLLFIAFLFSGIIYTYGHVLWK 215

                                          I3                                      TM6
sp|P21554|CNR1_HUMAN      AHSHAVRMIQRGTQKSIIIHTSEDGKVQVTRPDQARMDIRLAKTLVLILVVLIICWGPLL 360
sp|P34972|CNR2_HUMAN      AHQHVASLSGHQDR-------------QVPGMARMRLDVRLAKTLGLVLAVLLICWFPVL 262

                            TM6         E3            TM7                     H8
sp|P21554|CNR1_HUMAN      AIMVYDVFGKMNKLIKTVFAFCSMLCLLNSTVNPIIYALRSKDLRHAFRSMFPSCEGTAQ 420
sp|P34972|CNR2_HUMAN      ALMAHSLATTLSDQVKKAFAFCSMLCLINSMVNPVIYALRSGEIRSSAHHCLAHWKKCVR 322


sp|P21554|CNR1_HUMAN      PLDNSMGDSDCLHKHANNAASVHRAAESCIKSTVKIAKVTMSVSTDTSAEAL 472
sp|P34972|CNR2_HUMAN      GLG------------SEAKEEAPRSSVTETEADGKITPWPDSRDLDLSDC-- 360
```

MUTATIOS SITE 1:

F129L
I395V
F408A

MUTATIONS SITE 2:

C139Y
H154L
F237L

MUTATIONS SITE 3:

F191L
A198M
I245A

MUTATIONS SITE 4:

L193I
D366S
T377K

MUTATIONS SITE 5:

I243L
I247V
V282F

4. Liquid Chromatography/Mass Spectrometry (LC/MS) data

Supplementary Figure 5 | LC/MS analysis of ORG27569alkF3 treated CB_1. CB_1-wt HEK293 cells were treated with **ORG27569alkF3** (30 μM) for 24 hours to be then lysed. After immunoisolation, CB_1 was digested with Proteinase K and analysed by LC/MS. Comparison between **ORG27569alkF3** treated *vs.* untreated CB_1 MS spectra enables to identify peptides presenting the probe covalently attached ($\Delta M= + 447.8$ Da). a) HPLC profiles of **ORG27569alkF3** treated (green curve) and untreated samples (blue curve). Absorbance at $\lambda=254$ nm of the fractions (**ORG27569alkF3** (magenta curve) and untreated (red curve) samples) is reported. The dashed lines indicates the regions (retention time 3.8 and 14.0 min) where a difference in the HPLC profiles could be identified; the insets shows the MS spectra of sample fraction eluting at the two retention time and corresponding to CB_1 bound **ORG27569alkF3** (left panel) and unbound **ORG27569alkF3** (right panel); b) The 5 putative allosteric pockets mapped onto the CB_1 (accordingly to Fig.1a of the main text). The region addressed by **ORG27569alkF3** ($S^{2.45}$ and $S^{3.42}$) is depicted with red surface superimposed with the P2 binding pocket (cyan, surface). The orange spheres highlights other possible nucleophile sites (Ser, Thr, Cys, Tyr).

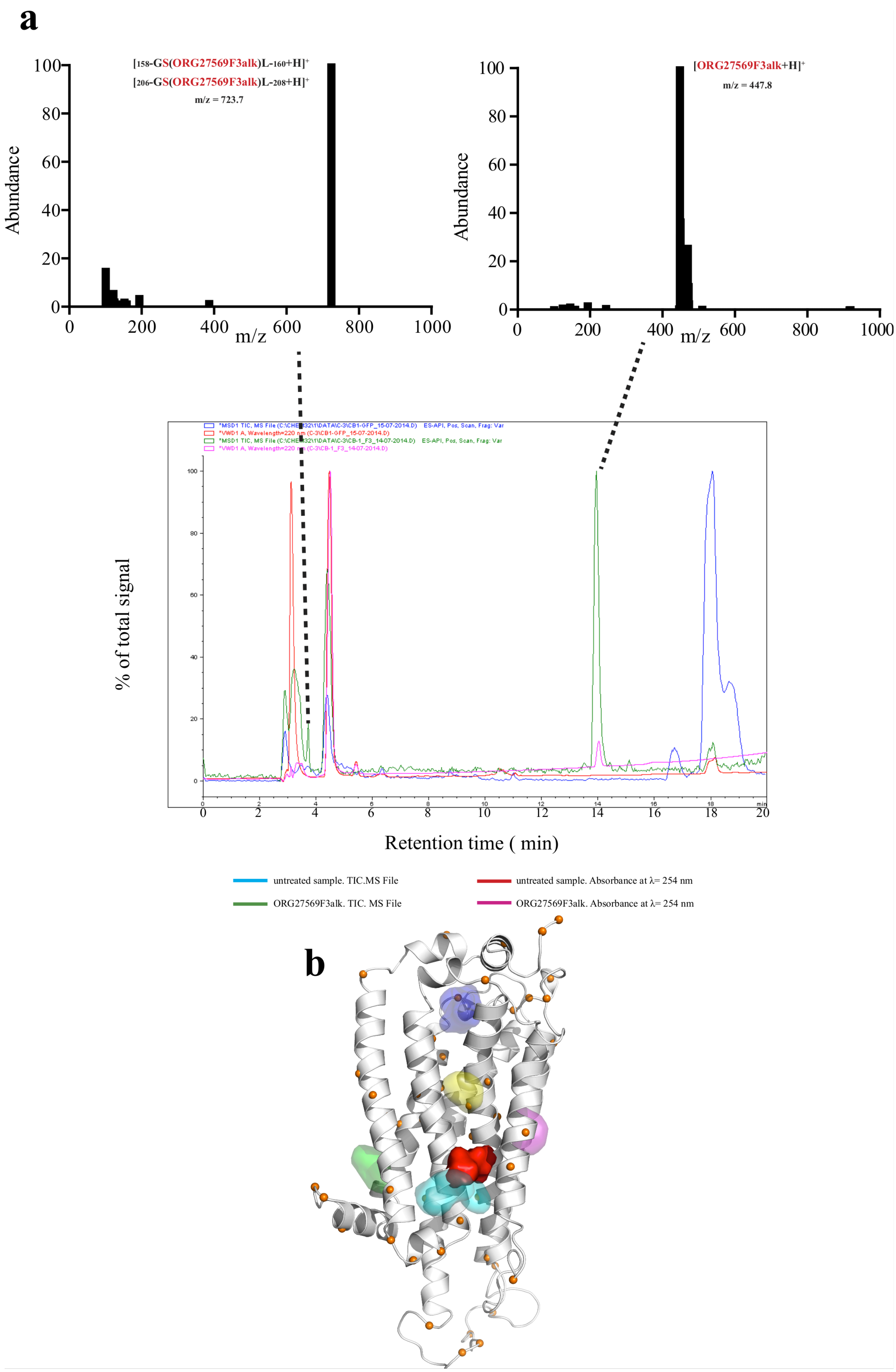
a
[158-GS(ORG27569F3alk)L-160+H]+
[206-GS(ORG27569F3alk)L-208+H]+
m/z = 723.7
Abundance
m/z
[ORG27569F3alk+H]+
m/z = 447.8
Abundance
m/z
% of total signal
Retention time (min)
untreated sample. TIC.MS File
untreated sample. Absorbance at λ= 254 nm
ORG27569F3alk. TIC. MS File
ORG27569F3alk. Absorbance at λ= 254 nm
b

Supplementary Figure 6 | Measurement of PM localization of CB1. a) Setting used to quantitate PM localization of CB1 and CB1-H 2.41L. N-terminally 3x Flag-CB1-GFP wt and mutant were transiently expressed in HuH7 cells. These were incubated after fixation (without permeabilization) with a rabbit polyclonal anti-FLAG antibody followed by a Texas-Red coniugated secondary antibody. The immunofluorescence intensity in the Texas-Red channel (depending only on the PM localized CB1) was measured using NIH ImageJ Biophotonic programs and normalized to one of the GFP channel (depending on the total CB1-GFP expression, PM+intracellular). For each transfection, 20 cells were considered for quantification. b) Percentage of cells showing CB1 and CB1 (H2.41L) localized at PM upon treatment with ORG27569 for 4 hours (data depict the mean +/- s.e.m. and are representative of three or more independent experiments . One-way ANOVA was employed. P<0.05).

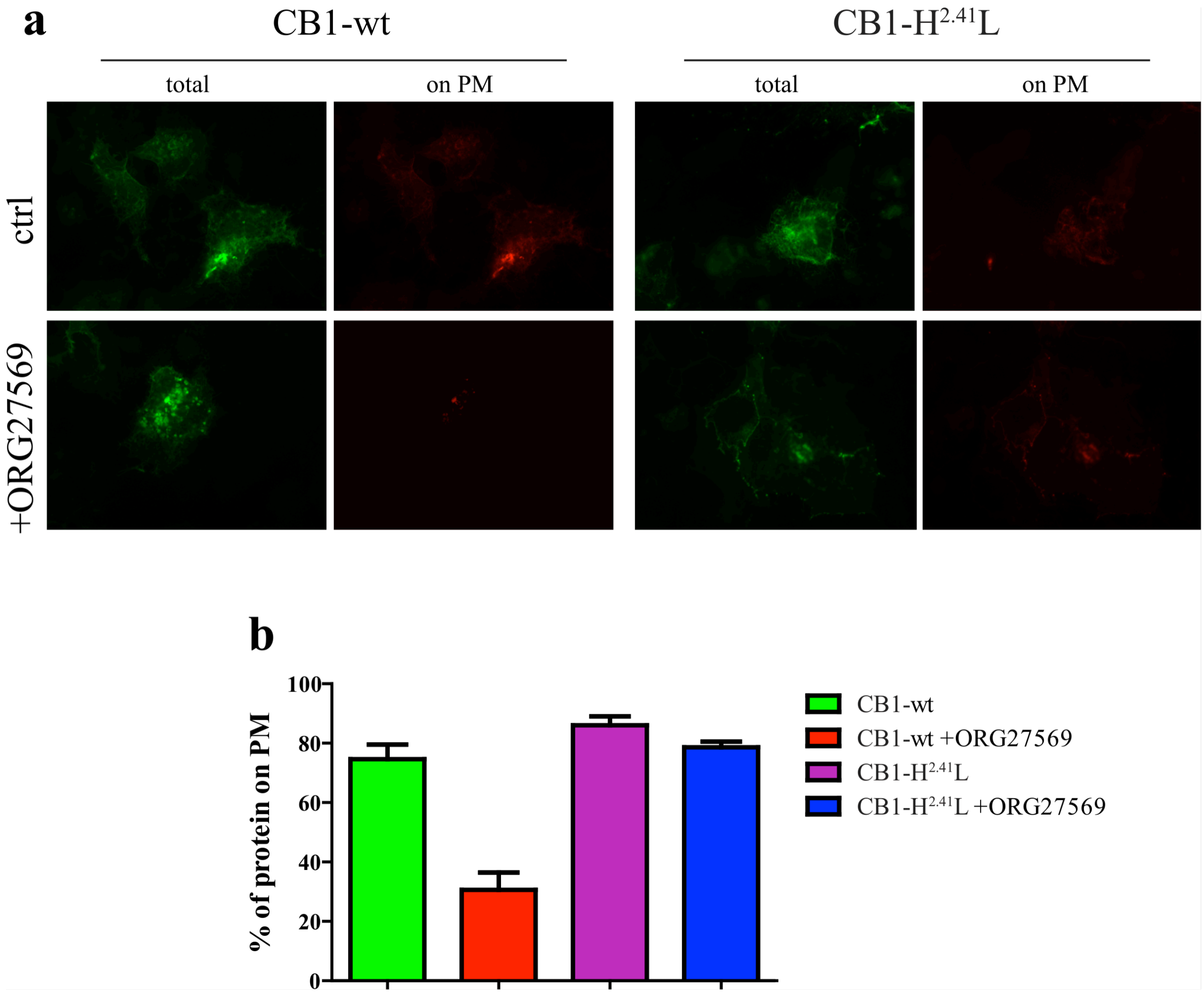

5. Binding mode reliability and analysis of the trajectories

Binding mode reliability

According to the binding studies on mutated CB_1 receptors, docking of ORG27569, by means of Glide software[11] was focused on P2 pocket. The resulting binding mode is depicted in Fig. 2a (main text), and the proposed pose fully explains the reported SAR[21]. In fact, the 3-ethyl chain, lying in a large apolar area, can be elongated without a loss of activity, the substitution of indole ring by a benzofuran moiety causes a reduced binding affinity due to the loss of the H-bond between the indole NH and the $H^{2.41}N\delta$ atom. The 1-(4-ethylphenyl)piperidine group protrudes toward the lipidic environment, establishing hydrophobic contacts with the receptor, accordingly to the reduced binding affinity observed for derivatives carrying hydrophilic substituents at this position.

Analysis of the trajectories

The PLUMED software[22] was used, and H-bonds (s) between specific donor-acceptor pairs were defined by a switching functions (eq.1), with r_0= 2.5, n=8 and m= 20. In Supplememntary Fig. 8 the probability of each H-bond is reported.

$$s = \sum_{ij} \frac{1 - \left(\frac{d_{ij}}{r_0}\right)^n}{1 - \left(\frac{d_{ij}}{r_0}\right)^m}$$

(eq.1)

Supplementary Figure 7 | Statistical analysis of the binding mode stability. (a) ORG27569 Binding mode in the first MD simulations, highlighted the H-bond interactions with $S^{2.45}$ (S1) and $H^{2.41}$(S2). (b) Comparison of the average binding modes of both run 1 and run 2 of CB_1wt-ORG27569 simulations (See Appendix A for the selection of the structures). Noteworthy, even starting from different binding conformations, the two CB_1wt-ORG27569 simulations seem to converge towards a unique binding mode. (c) Comparison of the average binding modes of both run 1 CB_1wt-ORG27569 and CB_1-($H^{2.41}$L)-ORG27569 simulations (See Appendix A for the selection of the structures). (d) Ligand RMSD for the run 1, run 2 CB_1wt-ORG27569 (black and red lines, respectively) and CB_1-($H^{2.41}$L)-ORG27569 (green line) simulations. (e, f) Probability distribution of the H-bond interactions between ORG27569 and $S^{2.45}$ and $H^{2.41}$ along the whole simulation of CB_1wt-ORG27569 system (run 1 and run 2). The plot highlights the region were both H-bonds are present (upper-right corner), were only one H-bond is present (lower-right and upper-left corners), and the region where no H-bonds were detected (lower-left corner). The probability of each state was normalized. H-bond interactions were computed as indicated in eq. 1.

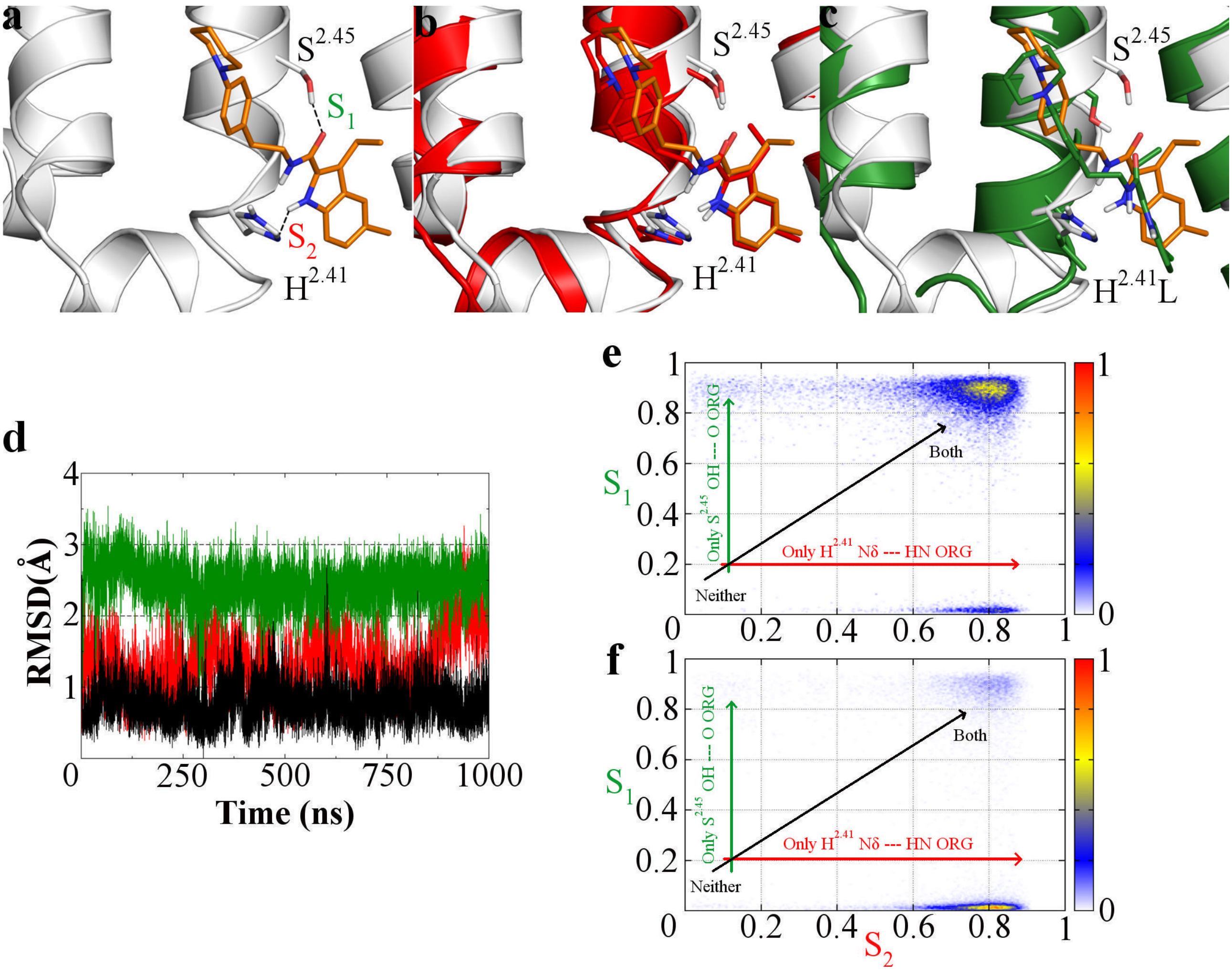

Supplementary Figure 8 | Statistical analysis of the structural effects induced by ORG27569 . (a) $H^{2.41}$ X_1 and X_2 dihedral angles distribution for the CB_1-wt simulations. (b) $H^{2.41}$ x_1 and x_2 dihedral angles distribution for the run 2 of the CB_1wt-ORG27569 simulation. A highly represented conformer, in the CB_1wt-ORG27569 MD, for the $H^{2.41}$ residue (conformer 2), different from that observed in the CB_1 unbound state (conformer 1, see also Fig. 2b and 2d in the main text) was noticed. Remarkably, in the CB_1wt-ORG27569 simulations, the shift from $H^{2.41}$ conformer 1 to 2 causes the H-bond loss between the $H^{2.41}$ Nε (TM2) and the R148 backbone oxygen (ICL1, Fig. 2d). The probability of each state was normalized. (c) Probability distribution for the $H^{2.41}$(NHε)-R148(O) distance atoms, for CB_1wt in POPC:Chol 2:1 membrane environment (blue lines), the CB_1wt-ORG27569 simulations (black , and red lines), and CB_1wt in DOPC:Chol 2:1 membrane environment (purple line), respectively. The probability of each distance was normalized. When CB_1 is embedded in a more flexible environment (DOPC) the probability distribution shows a second, less populated, pick around 5 Å. Noteworthy, in both CB_1wt-ORG27569 simulations a clear and well defined pick around 4 Å is appreciable, supporting the statistical robustness of this observation. This behavior is also highlighted when the average values for the $H^{2.41}$(NHε)-R148(O) distance are plotted with their standard deviation (d).

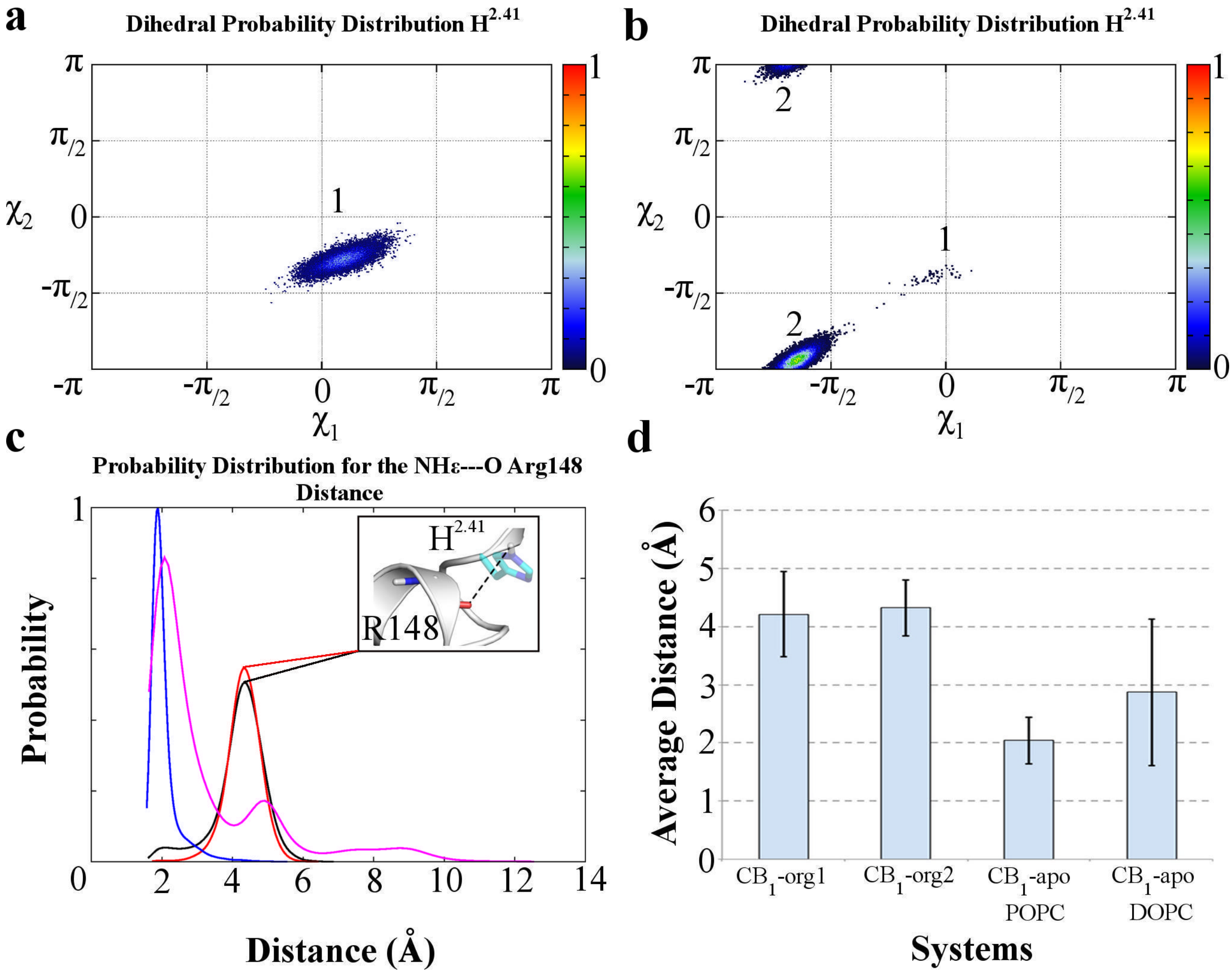

Statistical Analysis of ICL1-H8 conformations

The ICL1 and H8 conformations reported in Fig. 2a,d and h were obtained as described in Appendix A (Structure Selection). To compute the RMSD reported in Supplementary Figure 9a and b we used as reference structures for CB_1wt-ORG27569 and CB_1wt those reported in Figure 2a and h, respectively. The systems were aligned on the Cα atoms of the TM bundle, and the RMSD for the Cα atoms of the ICL1 and H8 domains was computed for the two CB_1wt-ORG27569 simulations [org1 (black line) and org2 (red line) in Supplementary Figure 9a,b], and for the CB_1wt simulations (blue line). From the analysis of the plots it is clear that the selected conformations (reported in Figure 2a,d and h) are stable under the simulations conditions. This trend is also highlighted by the calculations of the average RMSD for both ICL1 and H8 domains (Supplementary Figure 9c and d, respectively). Since, for all systems the average RMSD, with respect to the reference structure, is around 1.7 Å, which correspond to the canonical value for thermal fluctuation in classical MD simulations. Moreover, the similar behavior showed by the two CB_1wt-ORG27569 simulations for both ICL1 and H8 domains highlights how both systems converged toward similar conformations. Nevertheless, the RMSD only is not a sufficient parameters to describe how different is the conformational space explored by MD simulations, therefore we decided to compare CB_1wt-ORG27569 and CB_1wt simulations by performing a modified version of the Essential Dynamics Analysis, as reported below.

Essential Dynamics Analysis

The trajectories were analyzed with VMD[14,15]. An Essential Dynamics (ED) analysis[23] was performed by using the appropriate VMD plug-in in combination with the ptraj module of Amber11[16]. ED allows to identify a new essential subspace of coordinates by the diagonalisation of the covariance matrix of atomic fluctuations. For a X(t) multi-dimensioned system, where X could be a system of spatial coordinates, frame of molecular dynamics simulations etc., we could obtain the following covariance matrix:

$$C_{ij}=\left\langle \left(x_i-\left\langle x_i\right\rangle\right)\left(x_j-\left\langle x_j\right\rangle\right)\right\rangle$$

The symmetric matrix can be diagonalized by an orthonormal transformation matrix, which contains the eigenvectors or principal modes (columns of the matrix) and where the eigenvalues λ express the variance in the direction of the corresponding eigenvector. Every eigenvector is stored, in decreasing way, on the basis of the corresponding eigenvalue. Thus, for a dynamic process the

original data can be projected onto an eigenvector and the eigenvectors with larger eigenvalues match the more important displacement.

In our case a Combined Essential Dynamics (Comb-ED) analysis was performed[24]. In the Comb-ED the covariance matrix is calculated for two or more concatenated trajectories, and the overall translational and rotational motion are eliminated fitting all the trajectories on the same reference structure. First of all, both simulations of CB_1wt unbound and CB_1wt-ORG27569 were concatenated and clustered on the RMSD of the Cα atoms of the TM domains, using ptraj and the average-linkage cluster algorithm[16]. Ptraj turned out an average structure for the whole trajectories and a representative structure (the frame closest to the average structure in terms of RMSD). After that, both trajectories were finally fitted on the Cα atoms of the TM domains of the representative structure and the principal modes were computed for the Cα atoms of ICL1 (Supplementary Figure 9e) or H8 (Supplementary Figure 9e) only.

The analysis revealed, how ICL1 and H8 evolve in a different essential space (depending on the presence of ORG27569), and in turn, exploring different regions of the ICL1-H8 conformational space. The ICL1 and H8 starting conformation of all systems are the same (red box), since they were generated from the last steps of the initial 50ns simulations, for all systems.

Supplementary Figure 9 | Conformational Analysis of ICL1-H8 in the different simulations. (a) RMSD plot for ICL1 domain for the two CB_1wt-ORG27569 simulations [org1 (black line) and org2 (red line) in Supplementary Figure 9a,b], and for the CB_1wt simulations (blue line); (b) RMSD plot for H8 domain, color-coded as previously reported; (c) Average RMSD for ICL1 domain and H8 domain (d); (e) Comb-ED for ICL1 domain for CB_1wt-ORG27569 simulations (black line) and for the CB_1wt simulations (blue line); (f) Comb-ED for H8 domain for CB_1wt-ORG27569 simulations (black line) and for the CB_1wt simulations (blue line). Highlighted with a red box the location of the starting conformation of each domain and for each system.

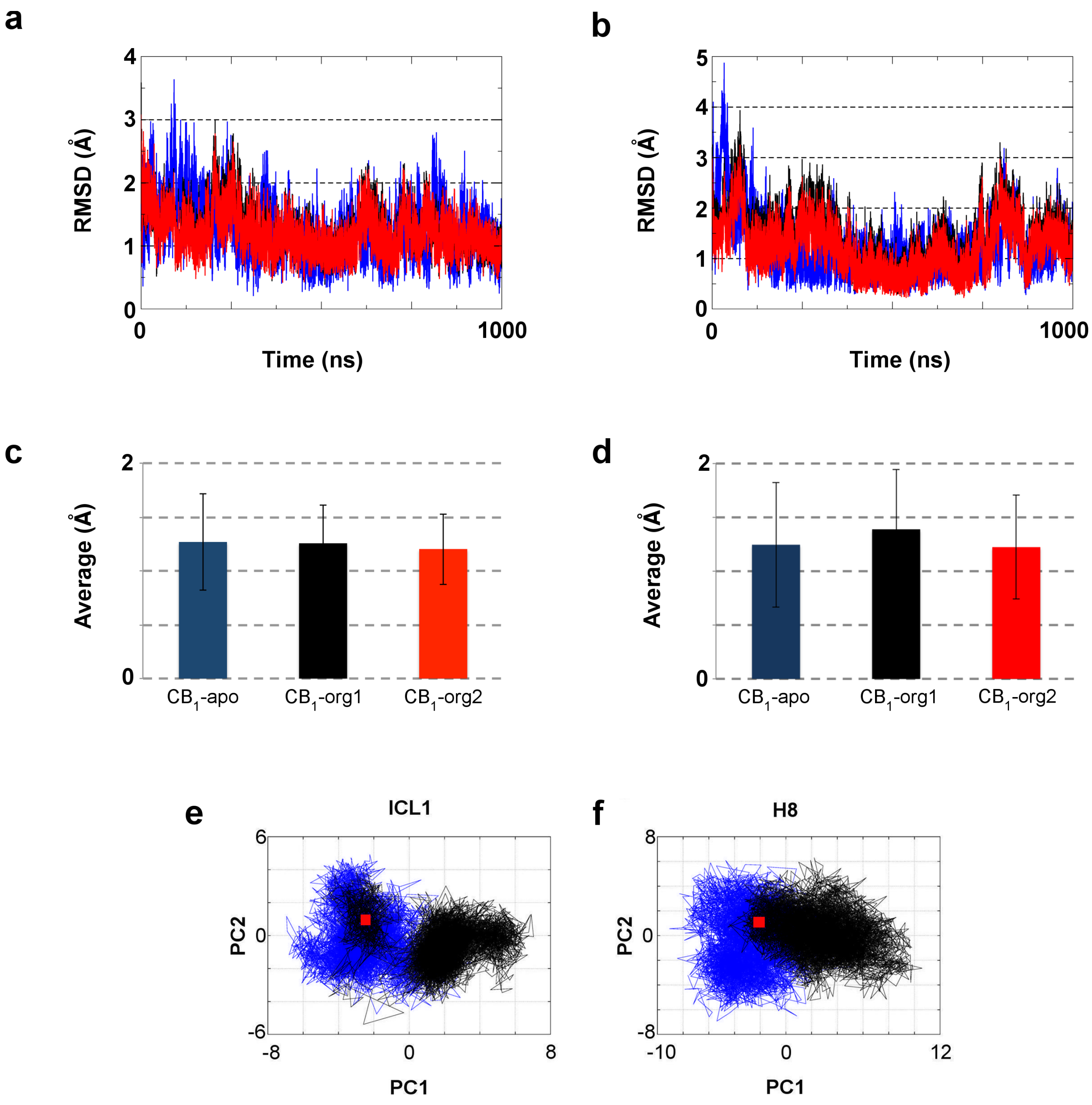

Supplementary Figure 10 | Statistical analysis of the TM3 effects induced by ORG27569. The comparison of the unbound CB_1wt and CB_1wt-ORG27569 simulations reveals a pronounced displacement of the TM3 C-terminus region, at the $T^{3.46}$ level (Supplementary Fig. S10a). This is another interesting result, since the region defined by $S^{2.45}$, $S^{3.42}$, $T^{3.46}$, $F^{2.}$42 has been implicated in the so called Hydrophobic Hindering Mechanism (HHM), in CB_1 and other GPCRs[25,26,27], supporting the idea that the alteration of this region can affect the orthosteric ligand binding affinity. (a) Comparison of the representative structures of both run 1 of CB_1-ORG27569 (white cartoon) and CB_1wt simulations (blue cartoon). Highlighted in stick and with the same color code the $S^{2.45}$ and $S^{3.42}$ residues, while with the same color code and sphere the Cα atoms of $F^{2.42}$ and $T^{3.46}$. The red arrow highlights the $T^{3.46}$ displacement. (b, c) Probability distribution for the R150(CZ)-$D^{8.49}$(CG) (b) and $F^{2.42}$(Cα)-$T^{3.46}$(Cα) (c) distance atoms, for CB_1wt (POPC:Chol 2:1) (blue lines), the CB_1wt-ORG27569 simulations (black , and red lines), the CB_1wt (DOPC:Chol 2:1) (purple line) and the CB_1-($H^{2.41}$L)-ORG27569 simulations (cyan line), respectively. (d) Together with the TM3 displacement, the presence of ORG27569 alters the pattern of H-bonds between $S^{2.45}$ and $S^{3.42}$. Indeed, in the CB_1wt simulations the $S^{2.45}$ and $S^{3.42}$ it breaks and it forms, periodically (blue line). In both run 1 and 2 of the CB_1wt-ORG27569 system the same interaction, after 450ns, is occasionally observed (black and red lines, respectively). (e) Average values for the R150(CZ)-$D^{8.49}$(CG) distance, with their standard deviation. (f) Average values for the $F^{2.42}$(Cα)-$T^{3.46}$(Cα) distances, with their standard deviation.

Of interest is the fact that in the CB_1-($H^{2.41}$L)-ORG27569 simulations these effects were not observed, although the presence of ORG27569 inside the P2 pocket (Supplementary Fig. 10b and c). These observation support the notion that, only the proper disposition of ORG27569 inside the P2 pocket induces the observed conformational changes, which are not observed when the anchoring $H^{2.41}$ is mutated in leucine (Supplementary Fig. 10b and c, cyan line).

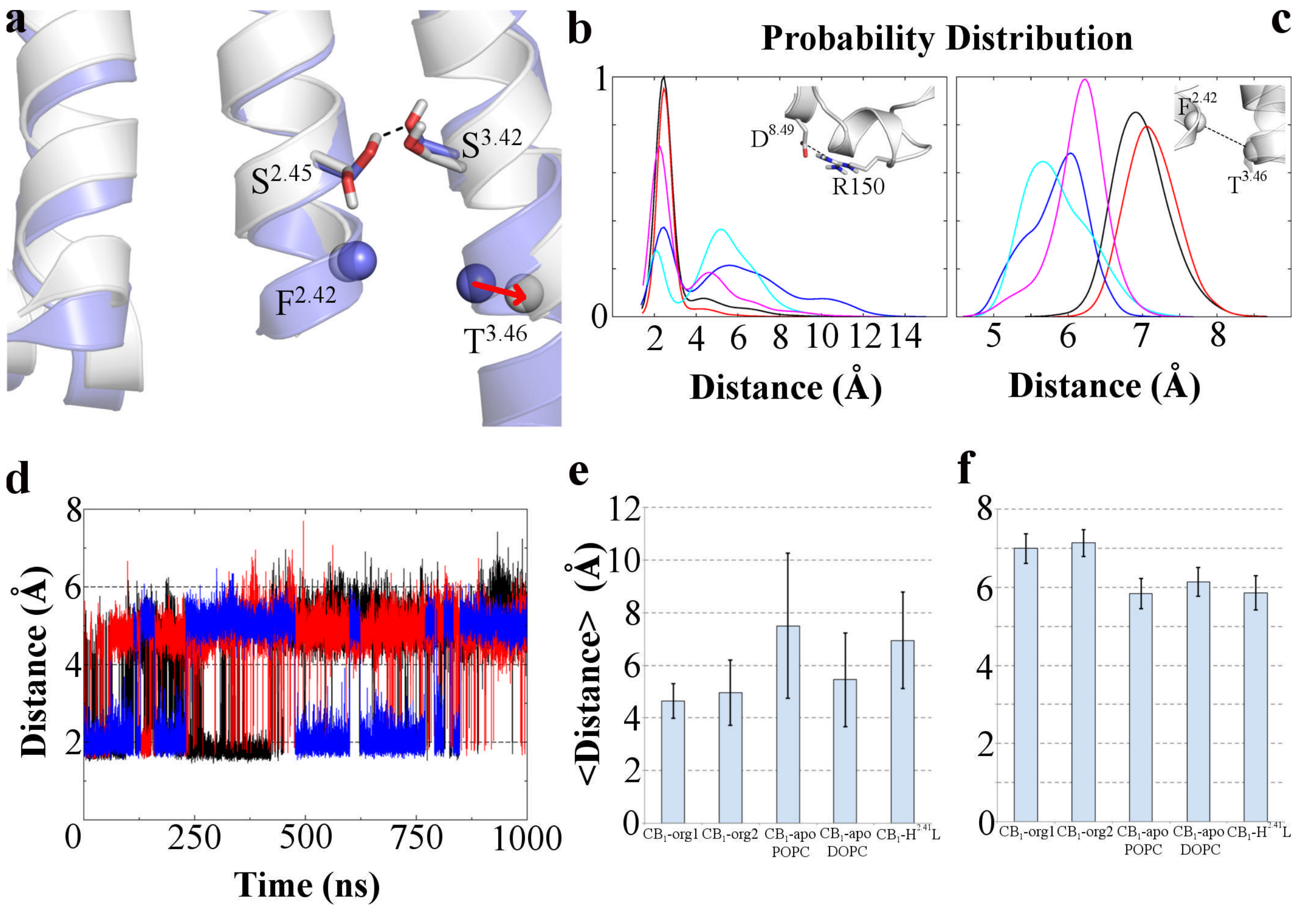

6. Analysis of GPCR X-ray crystal structures in complex with cholesterol, and cholesterol interaction with CB_1wt

Up to date about 120 GPCR X-ray crystal structures were disclosed for both family A, B, C and F, from different research groups. Among them 21 shows cholesterol molecules directly bound the TM bundle. In Supplementary Figure 10 we reported the main GPCRs families in complex with cholesterol. Noteworthy, it has been reported that cholesterol negatively affects the agonist binding for the β_2-adrenergic receptor. Indeed, cholesterol molecules bound to the receptor were found only in the inactive X-ray structures of such receptor (at the CCM level, PDB Code 2RH1 and 3D4S, Supplementary Figure 11a)[28], while no cholesterol molecules were found in the X-ray structures of the active state of the same receptor (PDB code 3PDS, 3P0G, 3SN6, 4LDO, 4LDL, 4LDE, 4QKX). On the contrary, it has been reported that platelet aggregation induced by P2Y12 activation (agonist mediated) is reduced after cholesterol depletion.[29,30] In the case of the P2Y12 receptor, cholesterol molecules were found in both the inactive and inactive states (Supplementary Figure 11e and f), but only in the active state a cholesterol molecule bound to the P2Y12-CCM was observed (Supplementary Figure 11f). On the basis of these experimental observation it can be hypothesized that CCM is one of the possible allosteric pockets, inside the GPCR families, through which cholesterol allosterically modulates GPCR functioning.

Supplementary Figure 11 | X-ray crystal structures analysis. GPCR X-ray crystal structures with cholesterol bound. **a.** β_2-adrenergic receptor (PDB code 3D4S). **b.** β_1-adrenergic receptor (PDB code 3ZPQ). **c.** 5-HT_{2B} receptor (PDB code 4IB4). **d.** A_{2A} receptor (PDB code 4EIY). **e.** Inactive P2Y12 receptor (PDB code 4NTJ). **f.** Active P2Y12 receptor (PDB code 4PXZ). **g.** μ-opioid receptor (PDB code 4DKL). **h.** mGlu1 receptor (PDB code 4OR2). Highlighted with a red circle cholesterol molecules bound to GPCR CCM.

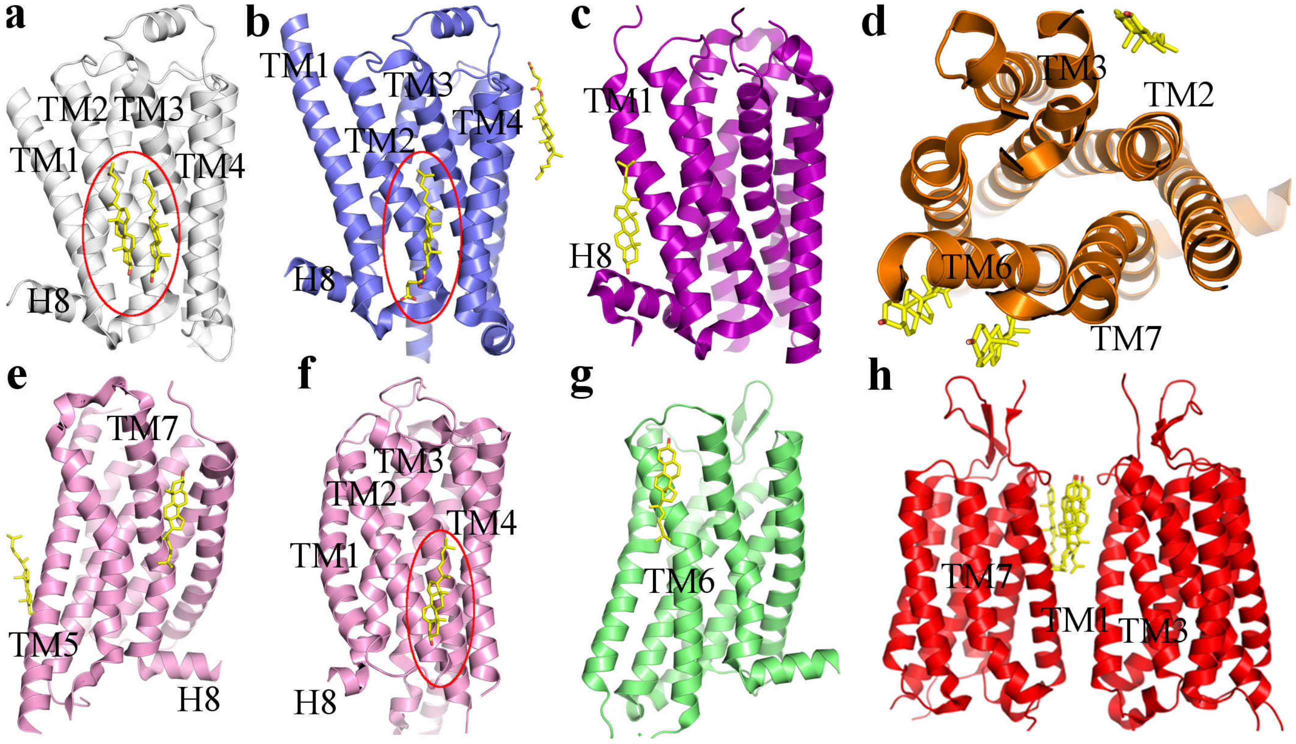

During the MD simulations of the CB_1wt systems cholesterol molecules interacts with different domains of the CB_1 receptor (Fig. 2f), including the P2 cleft (Fig. 2g and h and Supplementary Fig. S12), adopting a binding conformation similar to that observed in the CCM pocket of other GPCR X-ray structures (See Supplementary Fig. S11)[28].

Supplementary Figure 12 | Analysis of cholesterol binding in different membrane environment. a. Probability distribution of the cholesterol molecule, which binds to the CCM during the CB_1wt (POPC:Chol 2:1) simulations. **b.** Probability distribution of the cholesterol molecule in the CB_1wt (POPC:Chol 2:1 310K) simulations. **c.** Probability distribution of the cholesterol molecule in the CB_1wt (DOPC:Chol 2:1) simulations. The probability distribution distance for the $W^{4.50}$(Cα)-CHL(C13) carbon atoms (x axis) is plotted *vs.* the probability distribution distance for the NH(Arg148) and the O atoms (y axis). **d.** Binding conformation in the CB_1wt (POPC:Chol 2:1) simulations. **e.** Binding conformation in the CB_1wt (POPC:Chol 2:1 310K) simulations. **f.** Binding conformation in the CB_1wt (DOPC:Chol 2:1) simulations. **g.** Superposition of the three binding conformations obtained, in the different systems.

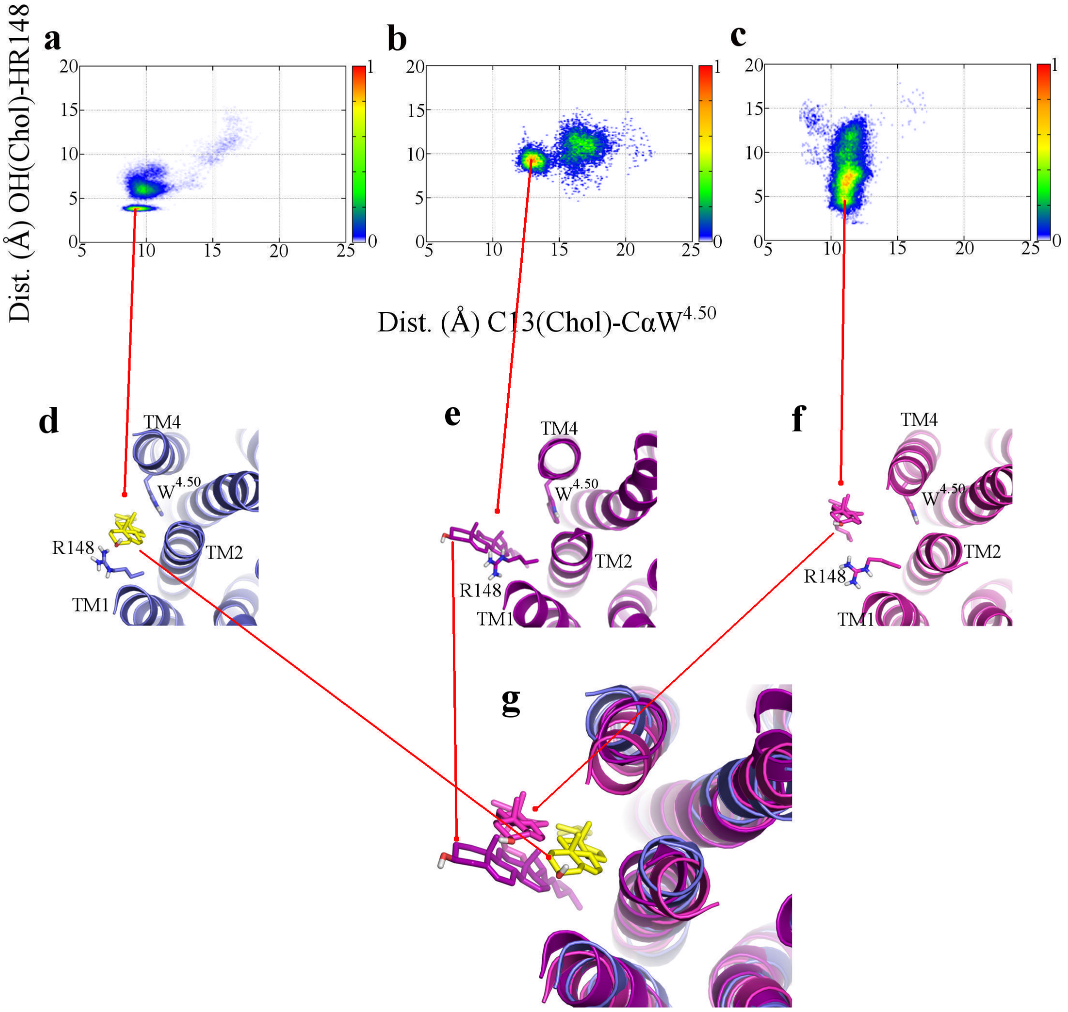

Supplementary Figure 13 | Cholesterol binding conformations. Superposition of the CB_1wt with a cholesterol molecule bound to the CCM (white cartoon and sticks), with the β_2-adrenergic receptor (PDB code 3D4S, yellow), β_1-adrenergic receptor (PDB code 3ZPQ, blue), and with the active P2Y12 receptor (PDB code 4PXZ, pink). The CB_1wt-cholesterol structure was obtained from the CB_1wt (POPC:Chol 2:1) simulations.

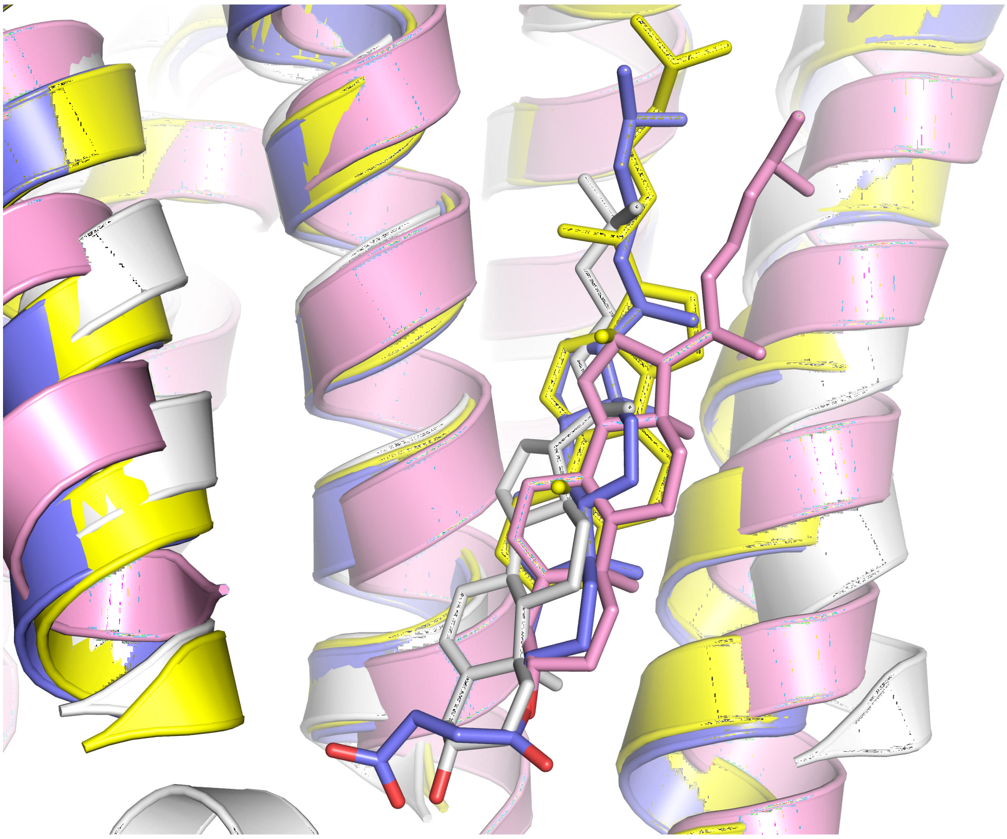

7. Generation of the $CB_1(H^{2.41}L)$-ORG27569 system.

Starting from the refined CB_1wt-ORG27569 complex $H^{2.41}$ was mutates in a leucine residue (Supplementary Fig. S14). The $CB_1(H^{2.41}L)$-ORG27569 complex, thus obtained, was submitted to the same MD protocol described for CB_1wt and CB_1wt-ORG27569 systems. A MD simulation 1 μs long was performed. For the CB_1wt-ORG27569 run 2 simulation, a different binding mode was selected (the 2 binding pose ranked in the docking studies) as starting point, and the system was submitted to the same MD protocol described for CB_1wt and CB_1wt-ORG27569 systems, and a MD simulation 1 μs long was performed.

Supplementary Figure 14 | Staring Conformations. Comparison of the CB_1wt-ORG27569 starting conformations (after minimizations) used for the MD simulations (cartoons and stick are depicted in white and blue, respectively for run 1 and run 2) and CB_1-($H^{2.41}L$)-ORG27569 (green) receptors in complex with ORG27569 (green sticks). In the figure ORG27569 and $H/L^{2.41}$ are depicted in the same color code, depending on the system considered: CB_1wt-ORG27569 run 1 (white), CB_1wt-ORG27569 run 2 (blue), CB_1-($H^{2.41}L$)-ORG27569 (green).

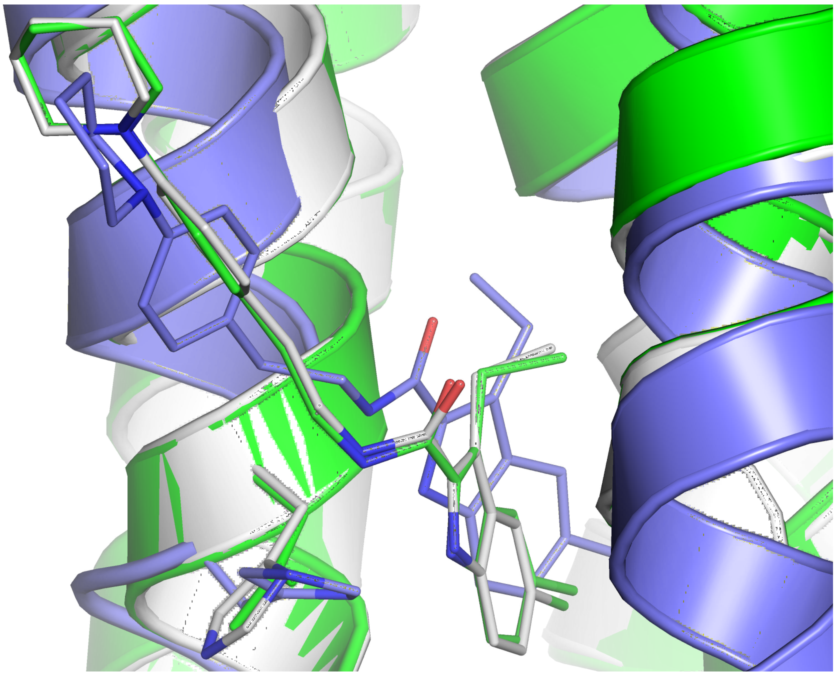

Supplementary Table 4 | MD simulations carried out. Sum up of the whole MD simulations carried out in this studiy.

System	Principal MD settings	Simulation Time
**CB_1wt-ORG27569 run 1**	Starting from the selected Docking pose	1 μs
**CB_1wt-ORG27569 run 2**	Starting from a different binding pose as depicted in Supplementary Figure S14	1 μs
**CB_1wt-apo 1**	POPC:Chol 2:1	1 μs
**CB_1wt-apo 2**	DOPC:Chol 2:1	1 μs
**CB_1wt-apo 3**	POPC:Chol 2:1<br>Cholesterol molecules with their Center Of Mass (COM) in close proximity to the CCM-COM (20 Å) were manually replaced with POPC. The MD simulations was conducted at 310K	1 μs
**CB_1-($H^{2.41}$L)-ORG27569**	$H^{2.41}$L mutation as depicted in Figure S14	1 μs

8. Appendix A.

Structure selection reported in the Figures of the manuscript and Supplementary Informations.

1) The structures of CB_1wt-ORG27569 reported in Figure 2 and Supplementary Fig. 7 are the representative structures obtained from the cluster analysis performed on the frames where ORG27569 forms two H-bonds with $H^{2.41}$ and $S^{2.45}$ (upper-right corner of the plot reported in Supplementary Fig. 5). Frames were clustered on the RMSD of the ORG27569 heavy atoms, using ptraj and the average-linkage cluster algorithm[16]. Ptraj turned out an average structure for the whole trajectories and a representative structure (the frame closest to the average structure in terms of RMSD).

2) The structure of the CB_1wt reported in Supplementary Figure 7 is the representative structure obtained from the cluster analysis performed on the whole CB_1wt simulations (1 μs). Frames were clustered on the RMSD of the Cα atoms of the TM domains, using ptraj and the average-linkage cluster algorithm[16]. Ptraj turned out an average structure for the whole trajectories and a representative structure (the frame closest to the average structure in terms of RMSD).

3) The structures of the CB_1wt interacting with cholesterol molecules reported in Figure 2 and Supplementary Figure 12 and 13 were selected taking into account only the structures showing the closest distance between the receptor and the cholesterol molecule.

Calculation of the Probability Distribution of Cholesterol on Figure 2f of the main text

The probability distribution of the cholesterol molecules in the lower leaflet was computed applying a protocol similar to that reported in *J. Am. Chem. Soc.*, **134**, 16512–16515, (2012). In Particular, only cholesterol molecules into the lower leaflet were considered. For each cholesterol molecules, the relative position of the center of mass along the x and y axes was computed with respect to the protein main axis (along z axis), as reported in Supplementary Figure 15.

Supplementary Figure 15 | Calculation of the Cholesterol Probability Distribution. Protocol used to compute the probability distribution function reported in Fig. 2f of the main text.

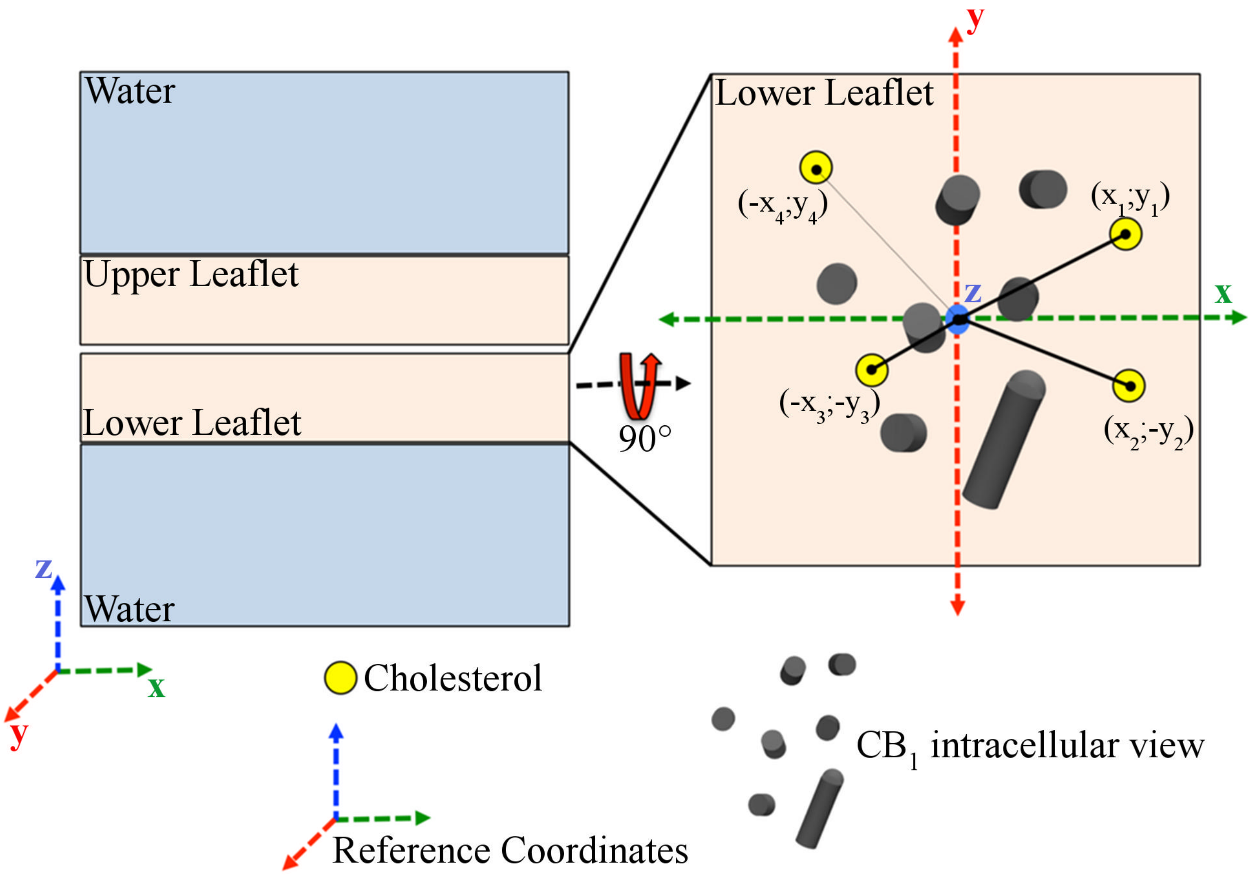

9. Chemistry section

General Methods. Commercially available reagents and solvents were used without further purification. Dichloromethane was dried by distillation from P_2O_5 and stored over activated molecular sieves (4 Å). When necessary the reactions were performed in oven-dried glassware under a positive pressure of dry argon. Melting points were determined in open glass capillaries and are uncorrected. All the compounds were characterized by IR. ^1H and ^{13}C NMR were recorded on a 400 MHz. High-resolution ESI-MS spectra were performed on a Thermo LTQ Orbitrap XL mass spectrometer. The spectra were recorded by infusion into the ESI source using MeOH as the solvent. Chemical shifts (δ) are reported in part per million (ppm) relative to the residual solvent peak. Column chromatography was performed on silica gel (70–230 mesh ASTM) using the reported eluents. Thin layer chromatography (TLC) was carried out on 5 x 20 cm plates with a layer thickness of 0.25 mm (Silica gel 60 F254). When necessary they were developed with $KMnO_4$.

Compound	Name	Structure
1	**ORG27569alk1**	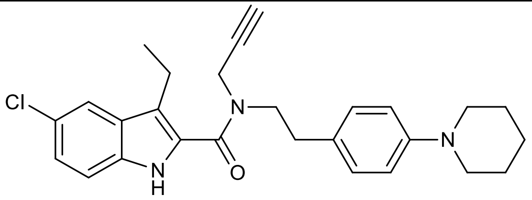
2	**ORG27569alk2**	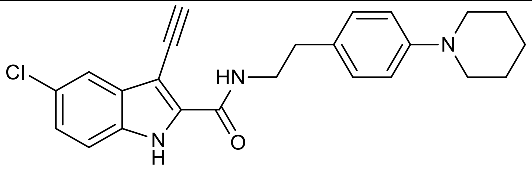
3	**ORG27569alk3**	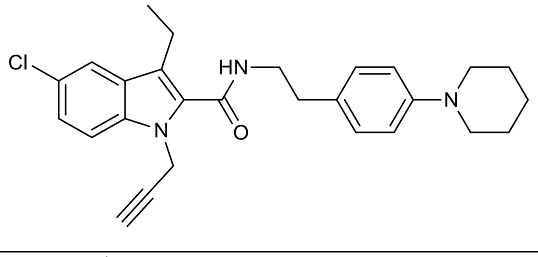
4	**ORG27569alk4**	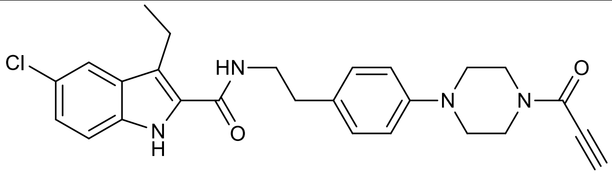

Procedures for ORG27569alk3

Supplementary Scheme 1

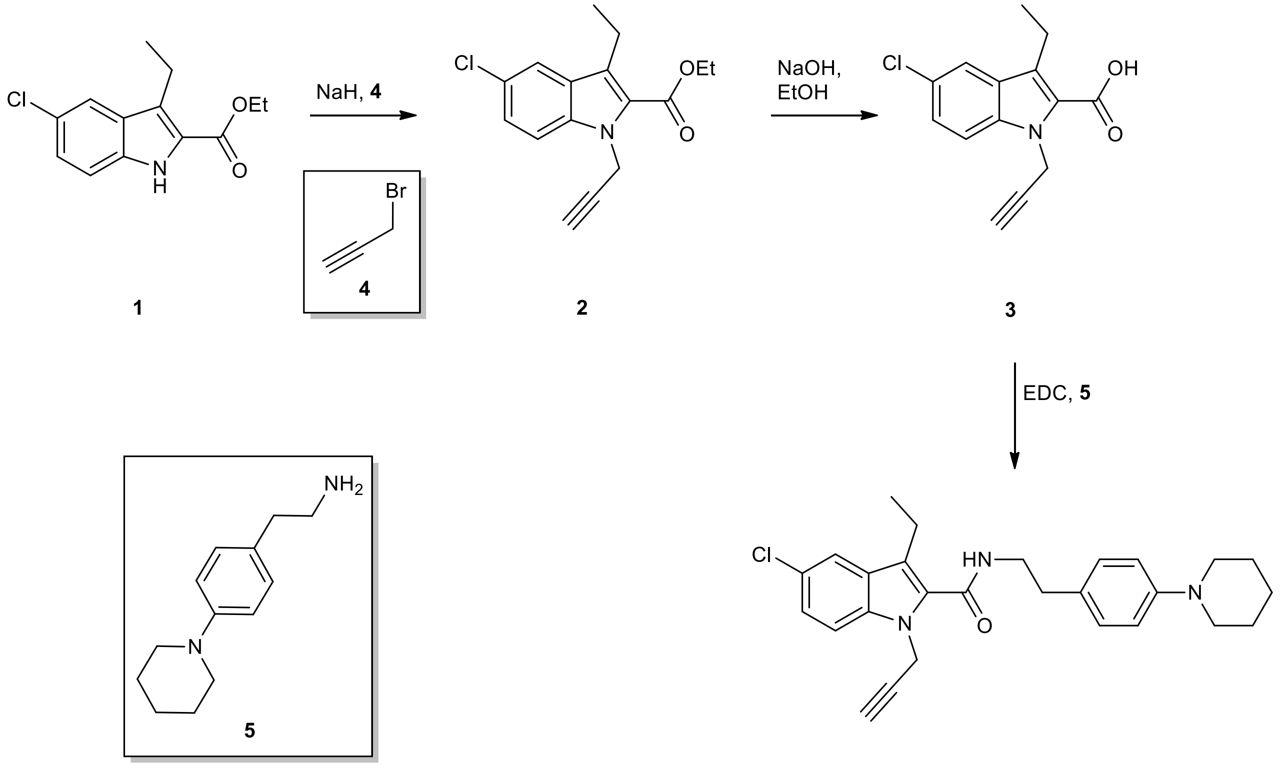

ORG27569alk3

Ester 2. To a solution of Ethyl 5-chloro-3-ethyl-1*H*-indole-2-carboxylate **1**[31] (2.18 g, 8.7 mmol) in dry DMF (20 mL) at 0 °C, was introduced sodium hydride (60% dispersion in mineral oil, 0.7 g, 17.4 mmol) and the mixture was stirred for 30 min at 0 °C. Propargyl bromide (0.95 mL, 10.4 mmol) was then added to the reaction mixture and reaction was stirred for 16 h at room temperature[32]. The crude reaction mixture was treated with water (100 ml) and the aqueous phase was extracted with Diethyl Ether (3 × 100 mL). The combined organic layers were dried over anhydrous $MgSO_4$ and the solvent was removed under reduced pressure. The crude product was purified by column chromatography on silica gel (EtOAc/ Hexane 1:5) to yield the substituted product **2** as a white solid (2.04 g, 81 %).

Mp: 107-109 ºC; TLC (EtOAc/ Hexane 1:3): R_f = 0.69; ^{1}H NMR (400MHz, CD_3OD): δ 7.67 (s, 1H), 7.50 (d, *J* = 8.96 Hz, 1H), 7.32 (dd, *J* = 6.96, 1.8 Hz, 1H), 5.36 (d, *J* = 2.16 Hz, 2H), 4.42 (q, *J* = 14.16, 7.04 Hz, 2H), 3.06 (q, *J* = 14.92, 7.48 Hz, 2H), 2.64-2.60 (m, 1H), 1.44 (t, *J* = 7.08 Hz, 3H), 1.24 (t, *J* = 7.44 Hz, 3H); ^{13}C NMR (100 MHz, $CDCl_3$): δ 162.3, 136.4, 127.9, 127.7, 126.1, 126.0, 124.3, 78.7, 71.9, 60.9, 34.4, 18.7, 15.5, 14.2; IR (neat) ν_{max}: 3274, 2967, 1695, 1267, 1144 cm-1; HRMS (ESI) $[M + H]^+$ calculated for $C_{16}H_{17}ClNO_2$: 290.048, found: 290.0933.

ORG27569alk3. A mixture of **2** (0.64 g, 2.22 mmol), 3 N sodium hydroxide (2.22 mL) and ethanol (5 mL) was heated to 60 °C for 1.5 h. After cooling, 1 N HCl was added until pH ~ 2. The mixture

was extracted with Ethyl Acetate (3 × 100 mL). The combined organic layers were dried over anhydrous $MgSO_4$ and the solvent was removed under reduced pressure. The carboxylic acid **3** was used for the next step without further purification.

EDC•HCl (0.065 g, 0.34 mmol) and 4-DMAP (0.041 g, 0.34 mmol) were added sequentially in single portions to a solution of carboxylic acid **3** (0.34 mmol) and amine **5**[31] (0.34 mmol) in dry DMF (5 mL) at room temperature. The mixture was stirred at room temperature for 16 h. Upon completion, the reaction contents were diluted with EtOAc (30 mL), poured into water (40 mL), and extracted with EtOAc (2 × 50 mL). The combined organic layers were then washed with 1 M aqueous HCl (3 × 30 mL) and brine (30 mL), dried ($MgSO_4$), and concentrated. The residue was purified by silica gel column chromatography (EtOAc/ Hexane 1:3) to provide **ORG27569alk3** as a white solid (0.84 g, 55 %).

Mp: 178-180 ºC dec.; TLC (EtOAc/ Hexane 1:3): $R_f = 0.29$; ^1H NMR (400MHz, $CDCl_3$): δ 7.55 (s, 1H), 7.33 (d, J = 8.8 Hz, 1H), 7.29-7.23 (m, 3H), 7.20-7.11 (m, 2H), 5.97-5.88 (m, 1H), 5.11 (d, J = 1.76 Hz, 2H), 3.77 (q, J = 12.32, 6.12 Hz, 2H), 3.24-3.07 (m, 4H), 2.90 (t, J = 5.4 Hz, 2H), 2.69 (q, J = 15.28, 7.64 Hz, 2H), 2.24 (s, 1H), 1.83-1.66 (m, 4H), 1.65-1.49 (m, 2H), 1.11 (t, J = 7.52 Hz, 3H); ^{13}C NMR (100 MHz, $CDCl_3$): δ 162.3, 135.4, 129.9, 129.8, 127.9, 125.9, 124.7, 119.6, 119.5, 78.6, 77.2, 72.4, 40.6, 34.6, 33.8, 18.03, 15.8; IR (neat) ν_{max}: 3285, 2928, 1621, 1544, 1454 cm-1; HRMS (ESI) $[M + H]^+$ calculated for $C_{27}H_{31}ClN_3O$: 448.2156, found: 448.2143.

Supplementary Scheme 2

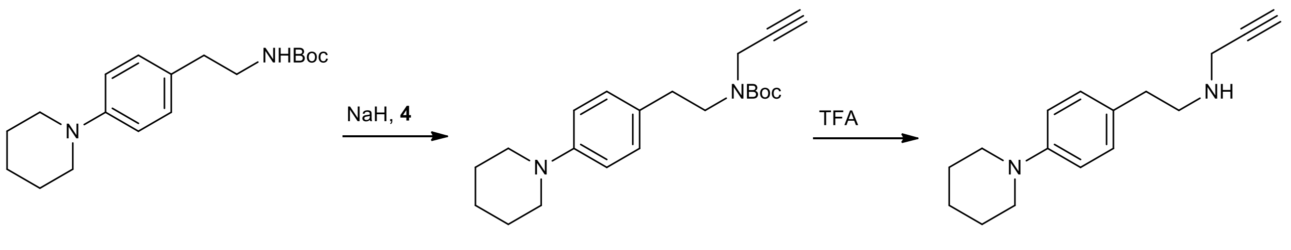

6 7 8

Procedures for compound 8

Carbamate 7. Boc-protected amine **6**[31] (0.216 g, 0.71 mmol) was dissolved in dry DMF (3 mL). The reaction mixture was cooled with an ice bath, and sodium hydride (60% in mineral oil, 0.035 g, 0.87 mmol) was added in small portions upon vigorous stirring. The reaction was allowed to proceed for 16 h at room temperature[33]. Then the reaction mixture was quenched with water (5 mL)

and extracted with Diethyl Ether (2 × 25 mL). The combined organic layers were dried over anhydrous $MgSO_4$ and the solvent was removed under reduced pressure. The crude product was purified by column chromatography on silica gel (EtOAc/ Hexane 1:7) to yield the substituted product **7** as a yellow oil (0.121 g, 47 %).

TLC (EtOAc/ Hexane 1:3): $R_f = 0.47$; ^{1}H NMR (400MHz, $CDCl_3$): δ 7.16-7.02 (m, 2H), 6.99-6.82 (m, 2H), 4.08-3.83 (m, 2H), 3.49 (t, $J = 7.4$ Hz, 2H), 3.19-3.04 (m, 4H), 2.79 (t, $J = 7.24$ Hz, 2H), 2.24-2.14 (m, 1H), 1.81-1.64 (m, 4H), 1.64-1.51 (m, 2H), 1.45 (s, 9H); ^{13}C NMR (100 MHz, $CDCl_3$): δ 154.6, 149.8, 129.4, 117.1, 80.1, 79.8, 77.2, 71.4, 51.2, 48.4, 36.1, 33.9, 33.6, 28.3, 25.7, 24.1; IR (neat) ν_{max}: 3307, 2928, 1695, 1511, 1160 cm-1; HRMS (ESI) $[M + H]^+$ calculated for $C_{21}H_{31}N_2O_2$: 343.2386, found: 343.2383.

Amine 8. A mixture of **7** (0.109 g, 0.3 mmol) and trifluoroacetic acid (0.3 mL) in dichlorometane (0.3 mL) was stirred at room temperature for 1 h. The solvent was evaporated and the residue was dried under high vacuum to provide crude **8** as bis- 2,2,2-trifluoroacetate which was used without further purification[31].

Procedures for ORG27569alk1

Supplementary Scheme 3

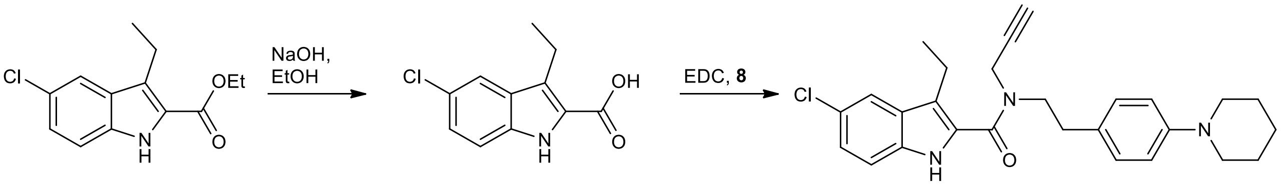

1 **9** **ORG27569alk1**

ORG27569alk1. A mixture of **1** (0.64 g, 2.22 mmol), 3 N sodium hydroxide (2.22 mL) and ethanol (5 mL) was heated to 60 °C for 1.5 h. After cooling, 1 N HCl was added until pH ~ 2. The mixture was extracted with Ethyl Acetate (3 × 100 mL). The combined organic layers were dried over anhydrous $MgSO_4$ and the solvent was removed under reduced pressure. The carboxylic acid **3** was used for the next step without further purification.

EDC•HCl (0.0658 g, 0.3 mmol) and 4-DMAP (0.036 g, 0.3 mmol) were added sequentially in single portions to a solution of the residue obtained above (0.3 mmol) and amine **8** (0.3 mmol) in dry DMF (5 mL) at room temperature. The mixture was stirred at room temperature for 16 h. Upon completion, the reaction contents were diluted with EtOAc (30 mL), poured into water (40 mL), and extracted with EtOAc (2 × 50 mL). The combined organic layers were then washed with 1 M

aqueous HCl (3 × 30 mL) and brine (30 mL), dried ($MgSO_4$), and concentrated. The residue was purified by silica gel column chromatography (EtOAc/ Hexane 1:3) to provide **ORG27569alk1** as a yellow oil (0.78 g, 58 %).

TLC (EtOAc/ Hexane 1:3): R_f = 0.28; ^1H NMR (400MHz, $CDCl_3$): δ 7.53 (s, 1H), 7.17-7.09 (m, 2H), 7.03-6.94 (m, 2H), 6.92-6.80 (m 2H), 4.35-4.18 (m, 2H), 3.76 (t, *J* = 5.88 Hz, 2H), 3.21-3.07 (m, 4H), 2.68 (q, *J* = 14.76, 7.12 Hz, 2H), 2.35 (s, 1H), 1.79-1.66 (m, 4H), 1.62-1.51 (m, 2H), 1.20 (t, *J* = 8.04 Hz, 3H); ^{13}C NMR (100 MHz, $CDCl_3$): δ 164.9, 134.0, 129.9, 128.0, 127.5, 125.3, 123.9, 119.4, 112.5, 78.5, 77.2, 73.1, 32.8, 29.7, 17.9, 15.0; IR (neat) ν_{max}: 3291, 2928, 1626, 1443, 1234 cm-1; HRMS (ESI) $[M + H]^+$ calculated for $C_{27}H_{31}ClN_3O$: 448.2156, found: 448.2143.

Procedures for ORG27569alk2

Supplementary Scheme 4

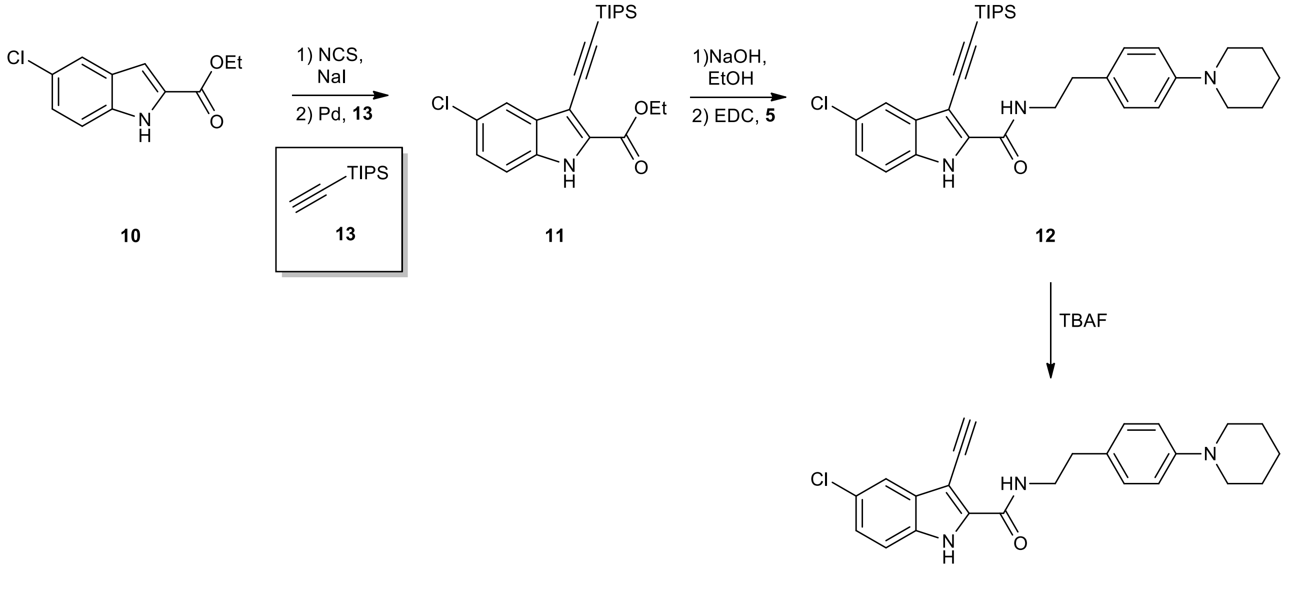

Alkyne 11. To a solution of N-chlorosuccinimide (1.59 g, 12 mmol) in DMF (25 mL) was added sodium iodide (1.79 g, 12 mmol) in small portions. The resulting brown solution was stirred at room temperature for 1 h before the slow addition at 0 °C of 5-chloro-1*H*-indole-2-carboxylate **10** (1.89 g, 10 mmol) in DMF (20 mL). The reaction mixture was stirred for 1 h at room temperature. A solution of sodium thiosulfate (10 %; 5 mL) and water (10 mL) were added and the mixture was stirred for 1 h. The solid was filtered, washed with cold water and cold hexane and dried under high vacuum[34]. The residue obtained was used for the next step without further purification.

To a mixture of the residue obtained above (0.525 g, 1.5 mmoL), $Pd(Ph_3P)_2Cl_2$ (0.053 g, 0.075 mmol, 5.0 mol %) and CuI (0.014 g, 0.075 mmol, 5.0 mol %) under argon atmosphere was added

triethylamine (7.5 mL) and stirred for 15 min. To this reaction mixture was added the terminal alkyne **13** (0.341 mL, 1.65 mmoL) and stirred at room temperature for 16 h. After completion, the reaction mixture was quenched with water (50 mL) and extracted with EtOAc (3 X 15 mL). The combined organic layers were dried over anhydrous $MgSO_4$ and the solvent was removed under reduced pressure. The crude product was purified by column chromatography on silica gel (EtOAc/ Hexane 1:10) to yield the product **11** as a white solid (0.429 g, 71 %)[35].

Mp: 136-138 ºC, TLC (EtOAc/ Hexane 1:3): $R_f = 0.52$; ^{1}H NMR (400MHz, $CDCl_3$): δ 9.24 (s, 1H), 7.72 (s, 1H), 7.36-2.27 (m, 2H), 4.47 (q, J = 14.16, 7.08 Hz, 2H), 1.73-1.63 (m, 3H), 1.43 (t, J = 7.08 Hz, 3H), 1.19 (s, 18H); ^{13}C NMR (100 MHz, $CDCl_3$): δ 161.1, 133.8, 130.3, 127.3, 126.7, 120.7, 113.4, 102.2, 102.0, 83.8, 75.7, 67.9, 61.7, 29.6, 25.5, 14.1 ; IR (neat) ν_{max}: 3313, 2939, 2587, 1684, 1253 cm-1; HRMS (ESI) $[M + H]^+$ calculated for $C_{22}H_{31}ClNO_2Si$: 404.1813, found: 404.1815.

TIPS Amide 12. A mixture of **11** (0.168 g, 0.42 mmol), 3 N sodium hydroxide (0.42 mL) and ethanol (1 mL) was heated to 60 °C for 1.5 h. After cooling, 1 N HCl was added until pH ~ 2. The mixture was extracted with Ethyl Acetate (3 × 25 mL). The combined organic layers were dried over anhydrous $MgSO_4$ and the solvent was removed under reduced pressure. The carboxylic acid **12** was used for the next step without further purification.

EDC•HCl (0.0658 g, 0.1 mmol) and 4-DMAP (0.036 g, 0.1 mmol) were added sequentially in single portions to a solution of the residue obtained above (0.1 mmol) and amine **5** (0.31 mmol) in dry DMF (2.8 mL) at room temperature. The mixture was stirred at room temperature for 16 h. Upon completion, the reaction contents were diluted with EtOAc (10 mL), poured into water (25 mL), and extracted with EtOAc (2 × 25 mL). The combined organic layers were then washed with 1 M aqueous HCl (3 × 10 mL) and brine (30 mL), dried ($MgSO_4$), and concentrated. The residue was purified by silica gel column chromatography (EtOAc/ Hexane 1:4) to provide **3** as a yellow solid (0.78 g, 40 %).

Mp: 183-185 ºC dec.; TLC (EtOAc/ Hexane 1:3): $R_f = 0.43$; ^{1}H NMR (400MHz, $CDCl_3$): δ 7.97-7.89 (m, 1H), 7.64 (s, 1H), 7.38 (d, J = 8.68 Hz, 1 H), 7.29-7.27 (m, 1H), 7.26-7.24 (m, 2H), 7.19-7.09 (m, 2H), 3.68 (q, J = 14.00, 6.32 Hz, 2H), 3.22-3.05 (m, 4H), 2.88 (t, J = 8.52 Hz, 2H), 1.85-1.64 (m, 4H), 1.63-1.49 (m, 2H), 1.29-1.23 (m, 3H), 1.17 (s, 18H); ^{13}C NMR (100 MHz, $CDCl_3$): δ 160.7, 133.8, 133.5, 130.2, 129.3, 127.2, 125.8, 120.3, 117.0, 113.8, 100.2, 99.5, 97.3, 44.8, 35.5, 25.7, 18.8, 11.3; IR (neat) ν_{max}: 3214, 2934, 1643, 1552, 1240 cm-1; HRMS (ESI) $[M + H]^+$ calculated for $C_{33}H_{45}ClN_3OSi$: 562.3020, found: 562.2985.

ORG27569alk2. Tetrabutylammonium fluoride (1 M in THF, 0.16 mL, 0.16 mmol) was added to a stirring solution of **12** (0.045 g, 0.08 mmol) in THF (1.2 mL). The reaction was stirred at room temperature for 16 h[34]. The reaction was quenched with a saturated solution of NH_4Cl (10 mL) and extracted with Diethyl Ether (3 X 10 mL). The organic layers were combined, washed with saturated $NaHCO_3$ (20 mL), brine (20 mL), dried with $MgSO_4$ and concentrated under reduced pressure. The crude mixture was purified by flash chromatography (EtOAc/ Hexane 1:3) to afford **ORG27569alk2** as a white solid (0.029 g, 90%).

Mp: 188-190 ºC dec.; TLC (EtOAc/ Hexane 1:3): $R_f = 0.36$; ^{1}H NMR (400MHz, $CDCl_3$): δ 7.68 (s, 1H), 7.61-7.53 (m, 1H), 7.38 (d, J = 8.68 Hz, 1H), 7.29-7.27 (m, 2H), 7.17 (d, J = 7.68 Hz, 1H), 7.06-6.91 (m, 2H), 3.81 (q, J = 12.72, 6.56 Hz, 2H), 3.39-3.33 (m, 1H), 3.21-3.10 (m, 4H), 2.89 (t, J = 6.64 Hz, 2 H), 1.83-1.70 (m, 4H), 1.68-1.51 (m, 5H); ^{13}C NMR (100 MHz, $CDCl_3$): δ 160.4, 134.2, 133.2, 129.8, 129.5, 127.1, 125.7, 125.4, 119.9, 117.5, 113.6, 95.5, 85.8, 76.3, 40.7, 34.4, 30.2, 29.6, 25.5, 23.8; IR (neat) ν_{max}: 3214, 3159, 1637, 1547, 1437 cm-1; HRMS (ESI) $[M + H]^+$ calculated for $C_{24}H_{25}ClN_3O$: 406.1686, found: 406.1681.

Procedures for ORG27569alk4

Supplementary Scheme 5

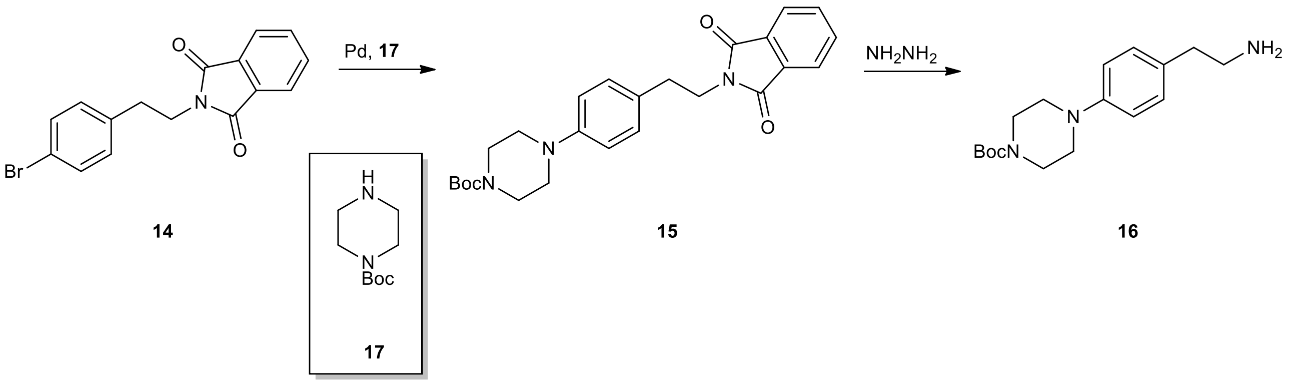

14 15 16 17

Phtalimide 17. A mixture of **14**[31] (0.263 g, 0.8 mmol), tris(dibenzylideneacetone)dipalladium(0) (0.022 g, 3.0 mol %, 0.018 mmol), XPhos (0.045 g, 0.096 mmol), cesium carbonate (0.652 g, 2.0 mmol) and piperidine **17**[35] (0.372 g, 2.0 mmol) in degassed anhydrous toluene (7 mL) was heated at 110 °C for 16 h[29]. After cooling, water and Diethyl Ether were added while shaking. The organic phase was washed with 3 N HCl (5 x 20 mL) and discarded. The acidic solution was neutralized with Na_2CO_3, and the mixture was extracted with Ethyl Acetate (3 X 25 mL). The combined organic layers were washed with brine, dried ($MgSO_4$) and evaporated. The crude mixture was

purified by flash chromatography (EtOAc/ Hexane 1:2) to afford **15** as a white solid (0.275 g, 79 %).

Mp: 166-168 ºC; TLC (EtOAc/ Hexane 1:3): R_f = 0.36; ^{1}H NMR (400MHz, $CDCl_3$): δ 7.82 (dd, J = 3.08, 2.32 Hz, 2H), 7.69 (dd, J = 3.04, 2.36 Hz, 2H), 7.16 (d, J =8.4 Hz, 2H), 6.85 (d, J = 7.38 Hz, 2H), 3.87 (t, J = 7.68 Hz, 2H), 3.61-3.52 (m, 4H), 3.12-3.04 (m, 4H), 2.91 (t, J = 8.0 Hz, 2H), 1.47 (s, 9H); ^{13}C NMR (100 MHz, $CDCl_3$): δ 168.1, 154.7, 149.9, 133.8, 132.1, 129.7, 129.6, 123.1, 120.2, 116.8, 79.8, 49.5, 39.3, 33.7, 30.78, 28.4, ; IR (neat) ν_{max}: 2934, 2851, 1711, 1681, 1544, 1454 cm-1; HRMS (ESI) $[M + H]^+$ calculated for $C_{25}H_{30}N_3O_4$: 436.2236, found: 436.2215.

Amine 16. A mixture of **17** (0.1 g, 0.3 mmol) and hydrazine monohydrate (0.075 g, 0.073 mL, 1.5 mmol) in ethanol (3 mL) was heated at reflux for 2 h[31]. After cooling, the reaction mixture treated with Diethyl Ether and 3 N HCl (5 x 20 mL) while shaking. The combined aqueous extracts were neutralized with Na_2CO_3 and extracted with Ethyl Acetate. The organic layers were washed with brine, dried ($MgSO_4$) and evaporated. The amine **18** was used for the next step without further purification.

Supplementary Scheme 6

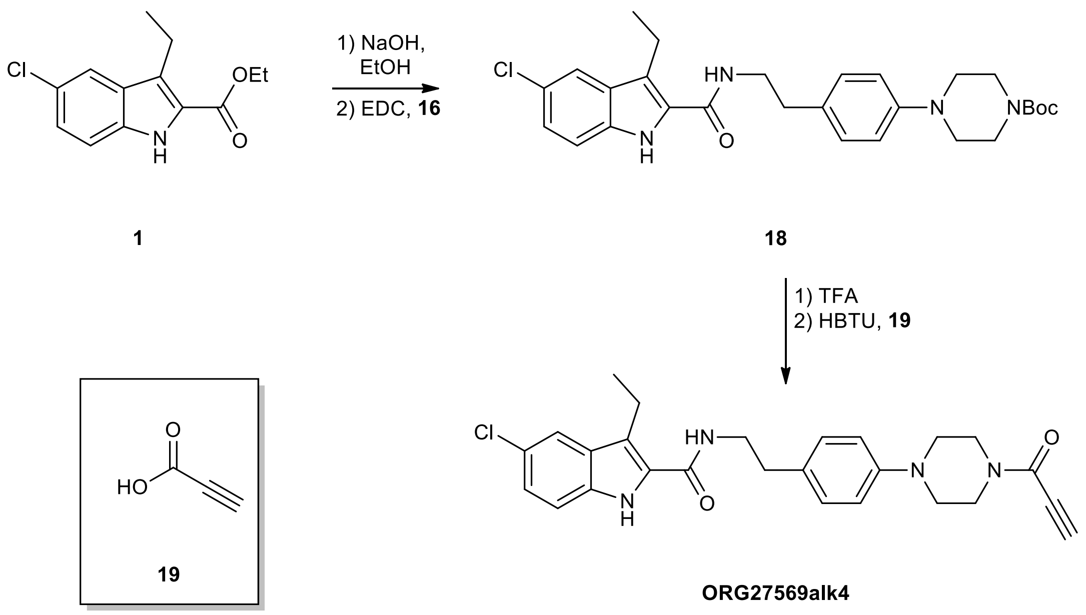

Amide 17. A mixture of **1** (0.64 g, 2.22 mmol), 3 N sodium hydroxide (2.22 mL) and ethanol (5 mL) was heated to 60 °C for 1.5 h. After cooling, 1 N HCl was added until pH ~ 2. The mixture was extracted with Ethyl Acetate (3 × 100 mL). The combined organic layers were dried over

anhydrous $MgSO_4$ and the solvent was removed under reduced pressure. The carboxylic acid was used for the next step without further purification.

EDC•HCl (0.0658 g, 0.3 mmol) and 4-DMAP (0.036 g, 0.3 mmol) were added sequentially in single portions to a solution of the residue obtained above (0.3 mmol) and amine **16** (0.3 mmol) in dry DMF (5 mL) at room temperature. The mixture was stirred at room temperature for 16 h. Upon completion, the reaction contents were diluted with EtOAc (30 mL), poured into water (40 mL), and extracted with EtOAc (2 × 50 mL). The combined organic layers were then washed with 1 M aqueous HCl (3 × 30 mL) and brine (30 mL), dried ($MgSO_4$), and concentrated. The residue was purified by silica gel column chromatography (EtOAc/ Hexane 1:3) to provide **3** as a yellow solid (0.78 g, 58 %).

Mp: 205-207 ºC dec.; TLC (EtOAc/ Hexane 1:3): R_f = 0.28; ^{1}H NMR (400MHz, $CDCl_3$): δ 9.22-9.14 (m, 1H), 7.54 (s, 1H), 7.29 (d, *J* = 8.68 Hz, 1H), 7.21 (d, *J* = 1.88 Hz, 1H), 7.20-7.14 (m, 2H), 6.99-6.90 (m, 2H), 6.03-5.95 (m, 1H), 3.78 (q, *J* = 12.32, 6.28 Hz, 2H), 3.67-3.54 (m, 4H), 3.17-3.06 (m, 4H), 2.90, (t, *J* = 6.56 Hz, 3H), 2.71 (q, *J* = 15.28, 7.64 Hz, 2H), 1.48 (s, 9H), 1.09 (t, *J* = 7.64 Hz, 3H); ^{13}C NMR (100 MHz, $CDCl_3$): δ 162.1, 135.8, 133.4, 129.6, 128.9, 128.2, 125.5, 124.9, 119.3, 112.9, 67.9, 40.8, 34.5, 34.2, 30.3, 29.7, 28.4, 21.2, 18.2, 15.3; IR (neat) ν_{max}: 3285, 2967, 1677, 1632, 1429 cm-1; HRMS (ESI) $[M + H]^+$ calculated for $C_{28}H_{36}ClN_4O_3$: 511.2476, found: 511.2441.

ORG27569alk4. A mixture of **18** (0.109 g, 0.3 mmol) and trifluoroacetic acid (0.3 mL) in dichlorometane (0.3 mL) was stirred at room temperature for 1 h. The solvent was evaporated and the residue was dried under high vacuum to provide the amine as bis- 2,2,2-trifluoroacetate which was used without further purification.

A mixture of the amine obtained above (0.005 mL, 0.08 mmol), Hunig base (0.021 mL, 0.12 mmol), **19** (0.08 mmol) and HBTU (0.036 g, 0.096 mmol) in dry DMF (0.220 mL) was stirred overnight at room temperature[38]. The reaction mixture was treated with H_2O (10 mL) and extracted with CH_2Cl_2 (2 X 25 mL). The combined organic layers was washed with brine, dried ($MgSO_4$) and concentrated. The residue was purified by silica gel column chromatography (EtOAc/ Hexane 1:3) to provide **23** as a yellow solid (0.015 g, 41 %).

Mp: 174-176 ºC dec.; TLC (EtOAc/ Hexane 1:3): R_f = 0.28; ^{1}H NMR (400MHz, $CDCl_3$): δ 9.14-9.07 (m, 1H), 7.55 (s, 1H), 7.29 (d, *J* = 8.72 Hz, 1H), 7.25-7.19 (m, 3H), 7.19-7.11 (m, 2H), 6.07-5.98 (m, 1H), 4.14-4.05 (m, 2H), 4.02-3.90 (m, 2H), 3.77 (q, *J* = 12.60, 6.56 Hz, 2H), 3.32-3.26 (m, 2H), 3.25-3.20 (m, 2H), 3.18 (s, 1H), 2.94 (t, *J* = 6.72 Hz, 2 H), 2.75 (q, *J* = 15.00, 7.40 Hz, 2H), 1.11 (t, *J* = 7.60 Hz, 3 H); ^{13}C NMR (100 MHz, $CDCl_3$): δ 160.4, 134.2, 133.2, 129.8, 129.5, 127.1,

125.7, 119.9, 117.5, 113.6, 95.6, 85.8, 76.1, 40.7, 34.4, 30.2, 29.6, 25.5, 23.9; IR (neat) ν_{max}: 3274, 2923, 1626, 1539, 1432 cm-1; HRMS (ESI) $[M + H]^+$ calculated for $C_{26}H_{28}ClN_4O_2$: 463.1901, found: 463.1904.

SUPPLEMENTARY METHODS

Mutagenesis. The following upstream (up) and downstream (dw) primers were used to introduce the indicated single amminoacidic sustituitions:

V282F-up gatgttctggatcgggTTCaccagcgtactgcttc
V282F-dw gaagcagtacgctggtGAAcccgatccagaacatc
T377K-up gaacaagctcattaagAAGgtgtttgcattctgc
T377K-dw gcagaatgcaaacacCTTcttaatgagcttgttc
L193I-up gtgtttctgttcaaaATCggtggggtcacggcctc
L193I-dw gaggccgtgaccccaccGATtttgaacagaaacac
I395V-up ctccaccgtgaaccccGTCatctatgctctgagg
I395V-dw cctcagagcatagatGACggggttcacggtggag
I247V-up gaccatagccattgtgGTCgccgtgctgcctctcc
I247V-dw ggagaggcagcacggcGACcacaatggctatggtc
I245A-up gatgtggaccatagccGCTgtgatcgccgtgctgc
I245A-dw gcagcacggcgatcacAGCggctatggtccacatc
I243L-up gcctgatgtggaccCTAgccattgtgatcgccgtg
I243L-dw cacggcgatcacaatggcTAGggtccacatcaggc
H154L-up gcaggccttcctacCTCttcatcggcagcctggcg
H154L-dw cgccaggctgccgatgaaGAGgtaggaaggcctgc
H154L-up (rat) gcaggccttcctacCTCttcatcggcagcctggc
H154L-dw (rat) gccaggctgccgatgaaGAGgtaggaaggcctgc
F408A-up gacctgcgacacgctGCCcggagcatgtttccctc
F408A-dw gagggaaacatgctccgGGCagcgtgtcgcaggtc
F237L-up caaggccgtggtggcgCTTtgcctgatgtggacc
F237L-dw ggtccacatcaggcaAAGcgccaccacggccttg
A198M-up ctgggtggggtcacgATGtccttcactgcctccg
A198M-dw cggaggcagtgaaggaCATcgtgaccccacccag
C139Y-up gaacctcctggtgctgTACgtcatcctccactccc
C139Y-dw gggagtggaggatgacGTAcagcaccaggaggttc

D366S-up gcaatcatggtgtatAGTgtctttgggaagatgaac

D366S-dw gttcatcttcccaaagacACTatacaccatgattgc

F129L-up cacgctgggcaccTTAacggtcctggagaacctcc

F129L-dw ggaggttctccaggaccgtTAAggtgcccagcgtg

F191L-up cgcaacgtgtttctgCTCaaactgggtgggg

F191L-dw ccccacccagtttGAGcagaaacacgttgcg

T1117 Fluorescent measurement. 50 µl of membrane suspension (15 to 30 µg/µl of total proteins) in PBS were incubated in 96 well black Optiplate (Perkin Elmer) with 500 nM T1117 (T1117 affinity binding) or with 3 µM T1117 (sensitivity to ORG27569). 15 minutes after the incubation the plates were inserted in a 2104 Envision Multi-label plate reader (Perkin Elmer). Non-specific binding was measured by adding AM251 2,5 µM to the sample and measuring change in T1117 fluorescence (specific quenching). Each sample was excited with Envision filter 206 (535 ± 25 nM ; 50% T) (Perkin Elmer) and fluorescence filtered with an emission filter 203 (615 ± 8.5 nM ; 80% T) (Perkin Elmer), using a normal top mirror. Plots were fitted in Prism5 (**GraphPad Software Inc**., La Jolla, CA) using inhibition sigmoidal curve to calculate IC_{50}.

Docking studies. The compound was geometrically optimized by means of Macromodel[11], using MMFFs as force field, water as implicit solvent until a convergence value of 0.05kcal/mol*$Å^2$. The computational protocol applied consist of the application of 500 steps of the Polak-Ribiére conjugate gradient (PRCG) for structure minimizations. The CB_1 protein structure was prepared through the Protein Preparation Wizard of the Maestro9.1[11]. Docking was accomplished through the Glide induced fit docking (IFD) tool available in Maestro9.1[11]. The grid was centered on the residues shaping the allosteric binding pocket defined by mutagenesis data reported in this work ($H^{2.41}$ and $F^{4.46}$ residues according to Ballesteros−Weinstein numbering). The flexible region of the protein was fixed until 8 Å around the center of the grid. Each docking run was carried out with the standard precision (SP) method, and the van deer Waals scaling factor of non polar atoms was set to 0.8. Fifteen docking poses were obtained and among these poses we selected the best pose in accordance with the mutagenesis data, and structure-activity relationship studies previously

reported for this class of CB_1 allosteric modulators[21]. The selected docking pose were minimized using OPLSA2005 as force field, the PRCG methods until a gradient of 0.001 kcal/mol*$Å^2$ applying a stepwise relaxation protocol for which harmonic constraints were progressively reduced for backbone, side chains and ligand atoms.

MD Simulations of CB1 unbound, CB1wt-ORG27569 bound and CB1-($H^{2.41}$L)-ORG27569 bound systems. The first CB1wt-ORG27569 bound form was built starting from the refined complex selected from the docking studies. ORG27569 atomic single charges were computed using Gaussian03, HF/6-31** as theory level (http://www.gaussian.com/g_misc/g03/g03_rel.htm#e1), and fitted with restrained electrostatic potential (RESP). The second CB1wt-ORG27569 bound form was built starting from a different binding conformation (the second ranked in the docking studies) and parametrized as described above (For details see Supplementary Fig. S14). Both CB1wt-ORG27569 complexes were modified as already described for the CB1wt unbound form (Protonation state, and palmitoylation site were considered as previously reported). The resulting complexes (CB1wt-ORG27569 x 2) were embedded in explicit POPC/Chol (2:1) membrane environment, while the CB1wt form was embedded in three different explicit membrane environments [POPC/Chol (2:1), DOPC/Chol(2:1), and POPC/Chol(2:1)-310K] as previously described, and the first N-terminal (S87) and the last C-terminal (E416) residues were capped with ACE and NME respectively. Only for the second POPC/Chol(2:1) system cholesterol molecules, having their center of mass (COM), in close proximity to the CCM-COM (20 Å) were replaced by POPC molecules, and the simulations was conducted at 310K for 1μs. Molecular dynamics simulations (relaxation and production phase) of the CB1wt-ORG27569 complexes were conducted as previously described for the CB1 unbound form. The final production phase was conducted for 1μs for each system.

The CB1wt-ORG27569 complex was used to generate the CB1-(H2.41L)-ORG27569 complex (Supplementary Fig. S14), and MD simulation 1μs long was conducted using the same MD setting reported previously. Computational and structural details about the generation of the different CB1 complexes and MD simulations setting are deeply discussed in Supplemental Material. Supplementary Table 4 lists all the MD simulations carried out.

SUPPLEMENTARY REFERENCES

1. Russ, A. P. & Lampel, S. The druggable genome: an update. *Drug Discov. Today* **10**, 1607–1610 (2005).

2. Oddi, S. *et al.* Effects of palmitoylation of Cys415 in helix 8 of the CB1 cannabinoid receptor on membrane localization and signalling: CB1 receptor palmitoylation. *Br. J. Pharmacol.* **165**, 2635–2651 (2012).

3. Salo, O. M. H., Lahtela-Kakkonen, M., Gynther, J., Järvinen, T. & Poso, A. Development of a 3D model for the human cannabinoid CB1 receptor. *J. Med. Chem.* **47**, 3048–3057 (2004).

4. Shim, J.-Y., Bertalovitz, A. C. & Kendall, D. A. Probing the Interaction of SR141716A with the CB1 Receptor. *J. Biol. Chem.* **287**, 38741–38754 (2012).

5. Martín-Couce, L. *et al.* Chemical Probes for the Recognition of Cannabinoid Receptors in Native Systems. *Angew. Chem. Int. Ed.* **51**, 6896–6899 (2012).

6. Hurst, D. P., Schmeisser, M. & Reggio, P. H. Endogenous lipid activated G protein-coupled receptors: Emerging structural features from crystallography and molecular dynamics simulations. *Chem. Phys. Lipids* **169**, 46–56 (2013).

7. ClustalW2 - Multiple Sequence Alignment at http://www.ebi.ac.uk/Tools/msa/clustalw2

8. Castillo, P. E., Younts, T. J., Chávez, A. E. & Hashimotodani, Y. Endocannabinoid Signaling and Synaptic Function. *Neuron* **76**, 70–81 (2012).

9. Paugh, S. W. Sphingosine and its analogue, the immunosuppressant fty720, interact with the cb1 cannabinoid receptor. *Mol. Pharmacol.* **70**, 41-50 (2006).

10. MODELLER, Program for Comparative Protein Structure Modelling by Satisfaction of Spatial Restraints at http://salilab.org/modeller

11. Schrödinger, Mestro version 9.1 Schrödinger, LLC, New York 2009 at http://www.schrodinger.com

12. Shim, J.-Y. Transmembrane Helical Domain of the Cannabinoid CB1 Receptor. *Biophys. J.* **96**, 3251–3262 (2009).

13. Ahn, K. H., Bertalovitz, A. C., Mierke, D. F. & Kendall, D. A. Dual Role of the Second Extracellular Loop of the Cannabinoid Receptor 1: Ligand Binding and Receptor Localization. *Mol. Pharmacol.* **76**, 833–842 (2009).

14. VMD - Visual Molecular Dynamics at http://www.ks.uiuc.edu/Research/vmd

15. Humphrey, W., Dalke, A. & Schulten, K. VMD: visual molecular dynamics. *J. Mol. Graph.* **14**, 33–38, 27–28 (1996).

16. The Amber Molecular Dynamics Package at http://ambermd.org

17. Ngan, C. H. *et al.* FTMAP: extended protein mapping with user-selected probe molecules.

Nucleic Acids Res. **40**, W271–275 (2012).

18. Ivetac, A. & Andrew McCammon, J. Mapping the Druggable Allosteric Space of G-Protein Coupled Receptors: a Fragment-Based Molecular Dynamics Approach: Computational Mapping of Novel Druggable Sites on GPCRs. *Chem. Biol. Drug Des.* **76**, 201-217 (2010).

19. Hendlich, M., Rippmann, F. & Barnickel, G. LIGSITE: automatic and efficient detection of potential small molecule-binding sites in proteins. *J. Mol. Graph. Model.* **15**, 359–363 (1997).

20. Laurie, A. T. R. & Jackson, R. M. Q-SiteFinder: an energy-based method for the prediction of protein-ligand binding sites. *Bioinformatics* **21**, 1908–1916 (2005).

21. Mahmoud, M. M. *et al.* Structure–Activity Relationship Study of Indole-2-carboxamides Identifies a Potent Allosteric Modulator for the Cannabinoid Receptor **1** (CB1). *J. Med. Chem.* **56**, 7965–7975 (2013).

22. Bonomi, M. *et al.* PLUMED: A portable plugin for free-energy calculations with molecular dynamics. *Comput. Phys. Commun.* **180**, 1961–1972 (2009).

23. Amadei, A., Linssen, A. B. & Berendsen, H. J. Essential dynamics of proteins. *Proteins* **17**, 412–425 (1993).

24. Shan, J., Khelashvili, G., Mondal, S., Mehler, E. L. & Weinstein, H. Ligand-Dependent Conformations and Dynamics of the Serotonin 5-HT2A Receptor Determine Its Activation and Membrane-Driven Oligomerization Properties. *PLoS Comput. Biol.* **8**, e1002473 (2012).

25. Ahn, K. H., Mahmoud, M. M. & Kendall, D. A. Allosteric Modulator ORG27569 Induces CB1 Cannabinoid Receptor High Affinity Agonist Binding State, Receptor Internalization, and Gi Protein-independent ERK1/2 Kinase Activation. *J. Biol. Chem.* **287,** 12070–12082 (2012).

26. Tehan, B. G., Bortolato, A., Blaney, F. E., Weir, M. P., Mason, J. S. Unifying family A GPCR theories of activation. *Pharmacol. Ther.* **143**,51-60, (2014).

27. Venkatakrishnan, A. J. *et al.* Molecular signatures of G-protein-coupled receptors. **Nature**, **494**, 185–194 (2013).

28. Hanson, M. A. *et al.* A Specific Cholesterol Binding Site Is Established by the 2.8 Å Structure of the Human beta2-Adrenergic Receptor. *Structure* **16**, 897–905, (2008).

29. Quinton ,T.M., Kim, S., Jin, J., Kunapuli, S.P. Lipid rafts are required in Galpha(i) signaling downstream of the P2Y12 receptor during ADP-mediated platelet activation. *J Thromb Haemost.* **3**, 1036-1041, (2005).

30. Savi, P. *et al.* The active metabolite of Clopidogrel disrupts P2Y12 receptor oligomers and partitions them out of lipid rafts. *Proc Natl Acad Sci U S A* **103**, 11069-11074, (2006).

31. Piscitelli, F. et al. Indole-2-carboxamides as allosteric modulators of the cannabinoid CB1 receptor. *J. Med. Chem.* **55**, 5627–5631 (2012).

32. Saptarshi De, James H. Rigby Total synthesis of (±)-debromoflustramine B via [4+1] cyclization of a bis(alkylthio)carbene and an indole isocyanate *Tetrahedron. Lett.* **54**, 4760-4762 (2013).

33. Denis S. Ermolat_ev, Jitender B. Bariwal, Hans P. L. Steenackers, Sigrid C. J. De Keersmaecker, and Erik V. Van der Eycken Concise and Diversity-Oriented Route toward Polysubstituted 2-Aminoimidazole Alkaloids and Their Analogues *Angew. Chem. Int. Ed. Engl.* **49**, 9465-9468 (2010).

34. Cyril Poriel, Mathilde Lachia, Claire Wilson, James R. Davies, and Christopher J. Moody Oxidative Rearrangement of Indoles: A New Approach to the EFHG-Tetracyclic Core of Diazonamide A *J. Org. Chem.* **72**, 2978-2987 (2006).

35. Chandrasekaran Praveen, Asairajan Ayyanar, Paramasivan Thirumalai Perumal Gold(III) chloride catalyzed regioselective synthesis of pyrano[3,4-b]indol-1(9H)-ones and evaluation of anticancer potential towards human cervix adenocarcinoma *Bioorg. Med. Chem. Lett.* **21**, 4170-4173 (2011).

36. Jonathan P. Brand, Julie Charpentier and Jérôme Waser Direct Alkynylation of Indole and Pyrrole Heterocycles An*gew. Chem. Int. Ed. Engl.* **48**, 9346-9349 (2009).

37. Chouaib Tahtaoui, et al Fluorescent Pirenzepine Derivatives as Potential Bitopic Ligands of the Human M1 Muscarinic Receptor *J. Med. Chem.* **47**, 4300-4315 (2004).

38. Muhammad Hashim Javaid, et al PARP inhibitors US 2006/0135770 Al
